# New tools for chloroplast genetic engineering allow the synthesis of human growth hormone in the green alga *Chlamydomonas reinhardtii*

**DOI:** 10.1007/s00253-016-7354-6

**Published:** 2016-02-18

**Authors:** Thanyanan Wannathong, Janet C. Waterhouse, Rosanna E. B. Young, Chloe K. Economou, Saul Purton

**Affiliations:** Algal Research Group, Institute of Structural and Molecular Biology, University College London, Gower Street, London, WC1E 6BT UK; Department of Biology, Faculty of Science, Silpakorn University, Nakornpathom, 73000 Thailand

**Keywords:** *Chlamydomonas*, Chloroplast, Genetic engineering, Human growth hormone

## Abstract

In recent years, there has been an increasing interest in the exploitation of microalgae in industrial biotechnology. Potentially, these phototrophic eukaryotes could be used for the low-cost synthesis of valuable recombinant products such as bioactive metabolites and therapeutic proteins. The algal chloroplast in particular represents an attractive target for such genetic engineering, both because it houses major metabolic pathways and because foreign genes can be targeted to specific loci within the chloroplast genome, resulting in high-level, stable expression. However, routine methods for chloroplast genetic engineering are currently available only for one species—*Chlamydomonas reinhardtii*—and even here, there are limitations to the existing technology, including the need for an expensive biolistic device for DNA delivery, the lack of robust expression vectors, and the undesirable use of antibiotic resistance markers. Here, we describe a new strain and vectors for targeted insertion of transgenes into a neutral chloroplast locus that (i) allow scar-less fusion of a transgenic coding sequence to the promoter/5′UTR element of the highly expressed endogenous genes *psaA* or *atpA*, (ii) employ the endogenous gene *psbH* as an effective but benign selectable marker, and (iii) ensure the successful integration of the transgene construct in all transformant lines. Transformation is achieved by a simple and cheap method of agitation of a DNA/cell suspension with glass beads, with selection based on the phototrophic rescue of a cell wall-deficient Δ*psbH* strain. We demonstrate the utility of these tools in the creation of a transgenic line that produces high levels of functional human growth hormone.

**Electronic supplementary material:**

The online version of this article (doi:10.1007/s00253-016-7354-6) contains supplementary material, which is available to authorized users.

## Introduction

Microalgae represent attractive biotechnology platforms for the synthesis of recombinant therapeutic proteins and other high-value products (Gong et al. 2011; Rasala and Mayfield 2015). Key advantages when compared to one or more of the traditional heterotrophic platforms such as *Escherichia coli*, yeast, or CHO cells include (i) the low-cost phototrophic cultivation of the alga in sterile, controlled photobioreactors using simple and inexpensive medium; (ii) the generally recognized as safe (GRAS) status of a number of algal species including the chlorophyte *Chlamydomonas reinhardtii*, making possible the oral delivery of bioactive products in whole algae and therefore avoiding expensive downstream purification; and (iii) the chloroplast organelle as a unique biosynthetic and storage compartment that contains its own minimal genetic system. Importantly, the use of the chloroplast for expression of foreign genes rather than the algal nucleus confers several benefits including precise insertion into the chloroplast genome (or “plastome”) via homologous recombination, high-level expression that is not subject to any gene-silencing mechanisms, the possibility of expressing multiple transgenes as operons, and accumulation of the recombinant proteins in a benign compartment where the formation of disulfide bonds occurs readily (Tran et al. 2009) but undesirable glycosylations are avoided (Gregory et al. 2013).

Chloroplast transgenics were first reported in 1991 when a bacterial antibiotic-resistance gene was expressed in *C. reinhardtii* (Goldschmidt-Clermont 1991). Since that time, there have been many reports describing the synthesis of functional therapeutic proteins in the *C. reinhardtii* chloroplast including monoclonal antibodies (Mayfield et al. 2003; Tran et al. 2009), growth factors (Rasala et al. 2010), antigens (Dreesen et al. 2010; Michelet et al. 2011; Jones et al. 2013), gut-active proteins (Manuell et al. 2007; Yoon et al. 2011), anti-bacterial proteins (Braun-Galleani et al. 2015), immunotoxins (Tran et al. 2013a, 2013b), and anti-toxins (Barrera et al. 2015). In addition, efforts are being made to manipulate chloroplast biosynthetic pathways in order to synthesize novel bioactive compounds such as diterpenoids (Gangl et al. 2015; Zedler et al. 2015). This encouraging progress in the development of the algal chloroplast as a viable platform has recently led to the establishment of start-up companies seeking to exploit the technology and the demonstration of pilot-scale production of a bioactive protein (Gimpel et al. 2015).

However, there remains a need to develop improved molecular tools that address some of the current technical limitations in the generation of *C. reinhardtii* transgenic lines (Purton et al. 2013). Specifically, there is a need for a simple and reliable method of rapidly generating homoplasmic transformant lines that also avoids the use of bacterial antibiotic-resistance genes as selectable markers. Currently, transformation typically involves bombardment of an algal lawn with DNA-coated microparticles (= biolistics) and the use of the *aadA* or *aphA6* bacterial genes as selectable markers conferring resistance to spectinomycin and kanamycin, respectively (Goldschmidt-Clermont 1991; Bateman and Purton 2000). Resistant colonies are then checked for the presence of the gene of interest (GOI) and taken through multiple rounds of single-colony selection in order to ensure that the transformant lines attain a stable, homoplasmic state in which all copies of the polyploid genome contain the marker and the GOI. A simpler alternative to microparticle bombardment involves agitating a suspension of cells and transforming DNA in the presence of glass beads, although this method requires the prior removal of the cell wall either by digestion or mutation (Kindle et al. 1991; Economou et al. 2014).

Similarly, one alternative method for selection employs non-photosynthetic mutants as recipient strains where the genetic lesion is in a key photosynthetic gene on the chloroplast genome. Selection is based on the use of a wild-type copy of the gene as the marker with successfully transformed cells able to grow phototrophically on minimal medium through replacement of the mutated gene with the wild-type version (Purton 2007; Michelet et al. 2011; Chen and Melis 2013). This selection strategy therefore allows the introduction of a GOI as the only transgene, avoiding the use of any antibiotic-resistance gene. Such “marker-free” transgenic lines are appropriate for industrial cultivation since they circumvent the regulatory and environmental issues associated with the possible horizontal transfer of such resistance genes into other microorganisms. Other issues associated with antibiotic-based selection include (i) the occurrence of “false-positive” colonies due to natural resistance mutations arising in *C. reinhardtii* genes, (ii) the additional metabolic burden on the chloroplast of replicating and expressing the marker, and (iii) the challenge of using antibiotic selection to drive transformant lines efficiently to a homoplasmic state.

Our recent work on *C. reinhardtii* chloroplast engineering (Braun-Galleani et al. 2015; Young and Purton 2014, 2016) has involved the use of a new recipient strain and expression vectors that allow the generation of marker-free transformants using the glass bead transformation method. In this paper, we describe these tools in detail. The recipient strain carries the nuclear *cw15* mutation resulting in a cell wall-deficient phenotype, together with a deletion of the essential photosystem II gene *psbH* to allow selection based on phototrophy. The expression vectors carry the *psbH* marker and are designed to allow simple cloning of a GOI as a perfect translational fusion with the promoter and 5′ untranslated region (UTR) from various chloroplast genes. We find that the vector that uses the *psaA* promoter/5′UTR element gives high expression levels in the chloroplast and is able to serve as a dual expression vector, since expression is also readily detectable in *E. coli*. Finally, we describe a simple three-primer PCR strategy to confirm both the correct integration of the GOI into the plastome and the establishment of homoplasmy. To validate these new tools, we have created a transgenic line expressing a synthetic gene encoding human growth hormone (hGH) and shown that the hGH is biologically active.

## Materials and methods

### *C. reinhardtii* strains and culture conditions

The TN72 strain is a *psbH*::*aadA* knockout mutant of the cell wall-deficient strain *cw15*.mt+ (Davies and Plaskitt 1971) and was created as described below. TN72 has been deposited in the publicly available collection of the Chlamydomonas Resource Center (chlamycollection.org) as strain CC-5168. TN72 transformant lines were initially selected on 2 % agar plates containing high salt minimal (HSM) medium but then maintained on Tris-acetate phosphate (TAP) agar plates (Harris et al. 2009) under dim light (5–10 μE/m^2^/s) at 20 °C. For liquid cultures, cells were grown in TAP medium in conical flasks at 25 °C, 120 rpm at 5 μE/m^2^/s (for TN72) or 50 μE/m^2^/s (for transformants). Spectinomycin (100 μg/ml) and erythromycin (10–50 μg/ml) were added to TAP agar medium as required.

### Plasmid construction

DNA manipulations were carried out using standard protocols (Green and Sambrook 2012), including transformation of chemically competent *E. coli* DH5α using ampicillin selection. Oligonucleotide primers were obtained from Eurofins Genomics (Ebersberg, Germany); restriction enzymes and T4 DNA ligase for cloning procedures were purchased from NEB (Ipswich, MA, USA) and Thermo Scientific (Waltham, MA, USA). Details of oligonucleotide primers used for plasmid construction are given in Table S1. Plasmid p72B-aadA-BM, which was used to create TN72, was made by replacing the 0.36-kb *Mlu*I-*Bst*XI region of p72B (Bateman and Purton 2000) with the “*aadA* cassette” (comprising the *aadA* coding sequence fused to the *atpA* promoter/5′UTR and *rbcL* 3′UTR) amplified from pUC-atpX-AAD (Goldschmidt-Clermont 1991) using primers that introduced an *Mlu*I and *Bst*XI site at the ends of the cassette. Transformation vector pASapI was created from p72B-SH (Bateman and Purton 2000), by first cutting p72B-SH with *Sap*I (within the pUC8 vector) and *Eco*RI, end-filling, and religating, thereby removing a 240-bp region of the vector sequence including the unwanted *Sap*I site. Then, the *Mlu*I-*Nco*I region containing the *rbcL* promoter/5′UTR element was replaced with an equivalent region amplified from *atpA* to give pASapI in which a *Sap*I site was created immediately downstream of the start codon. Vectors pSRSapI, pPSapI, and pCSapI were created from pASapI by replacing the *Mlu*I to *Sap*I region of pASapI with the promoter/5′UTR region of *psaA* exon 1, *psbA*, and *chlL*, respectively. The sequences of pASapI and pSRSapI are given in Fig. S1. The *ereB* coding sequence was amplified from plasmid pAT72 (Arthur and Courvalin 1986) using primers that introduce an *Nco*I and *Sph*I site at the start and end of the coding region, respectively, and cloned into these sites in pSRSapI to create pRY129a. The synthetic gene for hGH was codon-optimized for the *C. reinhardtii* chloroplast using the Codon Usage Database (www.kazusa.or.jp/codon) with *Sap*I and *Sph*I sites added for cloning (Fig. S2). The gene was synthesized by Life Technologies (Carlsbad, CA, USA) and cloned into the four expression vectors.

### Chloroplast transformation

Chloroplast transformation was based on a previously described method (Kindle et al. 1991) and involved the agitation of an algal/DNA suspension with glass beads of 400–625 μm diameter. A 400-ml culture grown to early log phase (approx. 2 × 10^6^ cells/ml) was concentrated by centrifugation and resuspended in TAP medium to 4 ml. Three hundred microliters of cells were added to a sterilized 5-ml test tube containing 300 mg sterile glass beads, followed by 5–10 μg circular plasmid DNA. The mixture was agitated vigorously at the maximum speed of a Vortex Genie II (Fisher Scientific, Loughborough, UK) for 15 s. The cells were spread on selective agar plates (TAP + spectinomycin at 100 μg/ml for *aadA* selection; HSM for *psbH* selection) after mixing with 0.5 % molten (42 °C) agar of the same selective medium. The plates were incubated at 25 °C in dim light (~2 μE/m^2^/s) overnight then transferred to a moderate light (~50 μE/m^2^/s) the next day. Transformant colonies were picked after 2–3 weeks and restreaked to single colonies several times on selective media to ensure homoplasmicity, although this was often achieved after the first streaking. Homoplasmy was determined by PCR using a combination of three primers (Table S1). Total genomic DNA was extracted from a loopful of cells using the Chelex 100 method as described by Werner and Mergenhagen (1998), and PCR amplification was carried out with Phusion DNA polymerase (Thermo Scientific) according to the manufacturer’s instructions. The photosystem II-deficient phenotype of TN72 was confirmed by measuring the photosynthetic capacity through chlorophyll fluorescence (Maxwell and Johnson 2000). Putative transformant lines were spotted onto TAP agar plates, incubated for 5 days and scanned using a pulse-modulated imaging fluorometer (FluorCam 700MF, Photon Systems Instruments, Czech Republic) as described by Wingler et al. (2004).

### Western analysis of *C. reinhardtii* and *E. coli* transformants

Protein samples from *C. reinhardtii* were prepared, fractionated on a 15 % acrylamide SDS-PAGE gel and blotted onto nitrocellulose membrane as described by Young and Purton (2014). For immunodetection, membranes were blocked in TBS-T (20 mM Tris, 137 mM NaCl, 1 M HCl pH 7.4, 0.1 % Tween-20) with 0.5 % milk powder overnight at 4 °C. Membranes were incubated with primary antibody in TBS-T with 0.5 % milk (1 h at RT), washed 3 × 5 min in TBS-T, incubated with secondary antibody in TBS-T with 0.5 % milk (1 h at RT), and washed as before. The probed blots were scanned and quantified using an Odyssey Infrared Imaging System (LI-COR). Alternatively, for Fig. 5a, the antibody binding was detected by enhanced chemiluminescence (ECL) using SuperSignal West Pico Chemiluminescent substrate (Thermo Scientific) and Hyperfilm ECL (GE Healthcare). For hGH detection, the primary antibody was chicken polyclonal antibody to human growth hormone (Abcam product ab27223) at 1:5000 dilution and the secondary was donkey anti-chicken IgY IRDye 800CW (LI-COR) at 1:5000, or goat anti-chicken IgY HRP-linked antibody (Abcam product ab97135) at 1:5000 for ECL. For RbcL detection, the primary antibody was anti-RbcL produced in rabbit (a gift from John Gray, Cambridge University, UK) at 1:5000 followed by secondary antibody Goat anti-Rabbit IgG (H&L), Dylight™ 800 Conjugated (Thermo Scientific product 35571) at 1:10,000, or HRP-linked Goat Anti-Rabbit IgG H&L (Abcam product ab6721) at 1:5000 for ECL.

For the detection of proteins in *E. coli*, 5 ml of an overnight LB culture was harvested by centrifugation (10 min at 4500 *g*) and resuspended in *X*/4 ml solution A (0.8 M Tris-HCl pH 8.3, 0.2 M sorbitol, 1 % β-mercaptoethanol) where *X* is the absorbance of the culture at 600 nm. SDS was added to a final concentration of 1 %. Samples were boiled for 5 min and passed through a 0.5-mm needle then centrifuged (2 min at 21,000 *g*), and 25 μl of supernatant was loaded. Gel and hGH immunodetection conditions using the ODYSSEY system were as described above. For both the *Chlamydomonas* and *E. coli* analysis, pure recombinant hGH (Abcam product 51232) was used as a reference.

### Nb2-11 cell proliferation assay for hGH activity

Nb2-11 cells (European Collection of Cell Cultures) were grown in suspension culture in Fischer’s medium supplemented with 10 % fetal bovine serum, 10 % horse serum, 2 mM l-glutamine, 0.075 % sodium bicarbonate, and 0.05 mM β-mercaptoethanol, at 37 °C in an atmosphere containing 5 % CO_2_. Cells were starved for 24 h by centrifugation (750 *g* for 5 min) to remove the culture medium, followed by washing and resuspending the cells in supplemented Fischer’s medium excluding fetal bovine serum. The cells were seeded in a 12-well plate at a concentration of approximately 1 × 10^5^ cells/ml. Recombinant hGH (Abcam) was added to 1 ml of cells in a volume of 5 μl to give a final concentration of 1, 10, or 100 ng/ml. Phosphate buffer (50 mM, pH 7.4) was added to cells as a control and was used to dilute the hGH. Samples were analyzed in triplicate. Cell proliferation was monitored every 24 h using the CASY^®^ Cell Counter and Analyzer Model TT (Innovatis, Bielefeld, Germany), with cells >8.03 μm being defined as viable. To test *C. reinhardtii* extracts, transformant TN-hGH, and the negative control TN-C, cells were cultured in 500-ml TAP medium to a concentration of 2 × 10^6^ cells/ml, broken by three cycles of freezing in liquid nitrogen and thawing, centrifuged at 9000 *g* for 30 min, followed by ultracentrifugation of the supernatant at 100,000 *g* for 60 min. The hGH content in the TN-hGH extract was quantified by Western blotting as above and a volume containing ~100 ng hGH used in the proliferation assay, together with an equivalent amount of TN-C extract, or phosphate buffer as controls. The experiment was repeated in triplicate with proliferation measured after 96 h.

### Sequence data

The Genbank accession number of the synthetic *hGH* used in the study is KU179444.

## Results

### The Δ*psbH* strain TN72 for reliable, marker-free selection of transformant lines

Our previous research has shown that disruption of the chloroplast gene *psbH*, which encodes the 9 kDa phosphoprotein subunit of photosystem II, results in a complete destabilization of this complex. As a result, *psbH* mutants are blocked in photosynthetic function and, thus, have an acetate-dependent phenotype (O’Connor et al. 1998). These mutants can be restored to phototrophy following biolistic transformation with a plasmid carrying wild-type *psbH*, with selection on minimal medium (Bateman and Purton 2000). The *psbH* gene can therefore be used as an effective selectable marker for *C. reinhardtii*, as has been established for other photosynthetic genes including *atpB*, *tscA*, and *rbcL* (Boynton et al. 1988; Goldschmidt-Clermont 1991; Chen and Melis 2013).

**Fig. 1:**
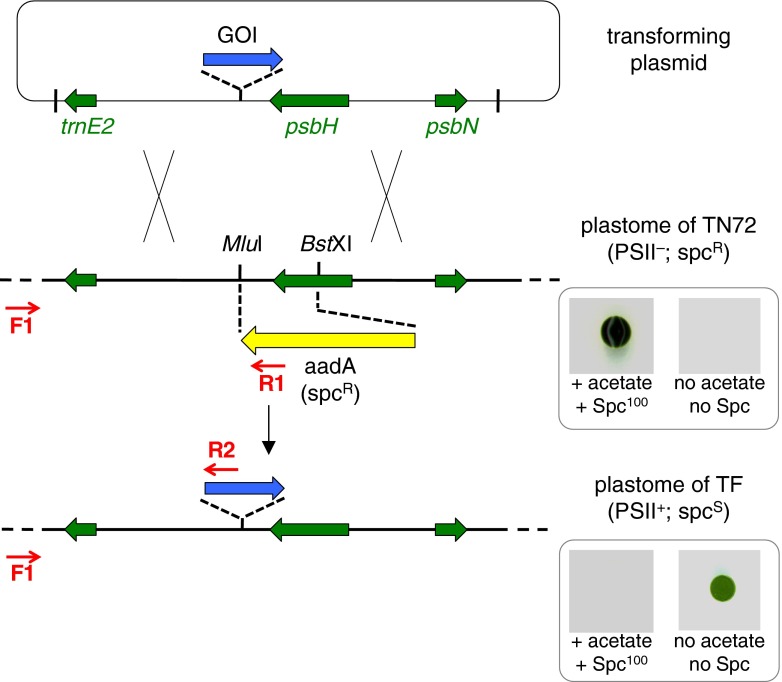
Strategy for generation of marker-less transformants using the TN72 recipient. The transforming plasmid contains the gene-of-interest (*GOI*) cassette which comprises a chloroplast promoter/5′UTR element from a chloroplast gene, coding sequence of the GOI and a 3′UTR from a chloroplast gene. The cassette is flanked by plastome sequence that includes *psbH* as selectable marker. Introduction into the chloroplast of strain TN72 (a knockout strain in which *psbH* and the downstream region have been replaced with the *aadA* marker) results in restoration of *psbH*, introduction of the GOI and loss of *aadA*. As a result, transformants (*TF*) are capable of phototrophic growth on acetate-free medium and are sensitive to spectinomycin (*Spc*) as shown in the *lower panel*. *Red arrows* indicate the three-primer set used to determine homoplasmy as detailed in Table S1

However, our original recipient strain “Bst-same” (O’Connor et al. 1998) contains a cell wall and therefore must be transformed either using the expensive biolistics method or by removing the cell wall using autolysin before glass bead-mediated transformation (Economou et al. 2014). To combine the benefits of photosynthetic selection and the simple, cheap glass bead method, a new *psbH* mutant (TN72) was created that carries the *cw15* nuclear mutation and thus lacks a cell wall. In addition, we eliminated one other flaw with Bst-same, which is that *psbH* is disrupted by insertion of the *aadA* marker into the coding region while GOIs are targeted to an intergenic site between *trnE2* and *psbH* that is 0.36 kb downstream of the *aadA* insertion. This means that transformation can occasionally generate transformants in which *psbH* is restored (and *aadA* eliminated), but the GOI is not introduced because homologous recombination between vector and genome has occurred in this 0.36 kb region. To solve this, the TN72 line was constructed such that the whole region is replaced by the *aadA* marker, thereby disrupting *psbH* and ensuring that transformants can arise only by integration of both *psbH* and the GOI (Fig. 1). Details of the creation of TN72 are given in Figs. S3 and S4.

### New expression vectors allow a perfect fusion of the GOI to the 5′UTR and rapid establishment of chloroplast homoplasmy

**Fig. 2:**
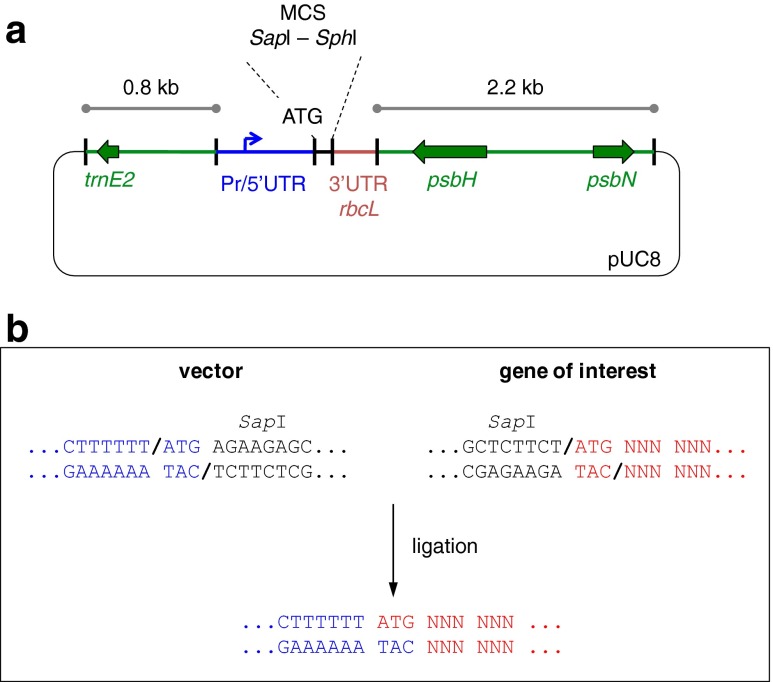
Design of expression vectors and *Sap*I cloning scheme. **a** Four different vectors were constructed that contained a promoter/5′untranslated region (*Pr/5′UTR*) from different chloroplast genes, namely pASapI (*atpA*), pSRSapI (*psaA*-exon 1), pPSapI (*psbA*), and pCSapI (*chlL*). **b** The positioning of the *Sap*I site in the vector and in any synthetic gene of interest to allow a perfect fusion at the ATG start codon

Translation is a key regulatory step in gene expression in the *C. reinhardtii* chloroplast (Eberhard et al. 2002) and typically involves one or more *trans*-acting protein factors that bind to *cis* elements with the 5′UTR of the transcript and mediate efficient ribosome binding and translation initiation (Choquet and Wollman 2002; Specht and Mayfield 2013). Furthermore, the bases immediately upstream of the start codon have been shown to play an important role in efficient translation initiation (Esposito et al. 2001). We therefore designed an expression vector that uses the type IIS restriction enzyme *Sap*I for cloning. As illustrated in Fig. 2, *Sap*I cuts to give a three-base 5′ overhang to one side of its recognition site. This allows the joining of a chloroplast promoter/5′UTR element to the ATG of a GOI as a perfect fusion without the incorporation of the site itself, and therefore without the need to introduce any DNA changes upstream of the start codon or add additional codons to the start of the GOI.

In order to test different transcription and translation efficiencies, four different versions of the vector were constructed that each have the same multiple cloning site for GOI insertion followed by the 3′UTR from the endogenous *rbcL* gene, but differ in the endogenous promoter/5′UTR element used to drive expression, namely that from *psaA* exon 1 (vector pSRSapI), *atpA* (pASapI), *psbA* (pPSapI), and *chlL* (pCSapI) as detailed in Table S2. Each vector can be used in combination with the TN72 line to target any GOI to the neutral locus within the *psbH-trnE* intergenic region, using restoration of *psbH* as selection as illustrated in Fig. 1.

Since the *C. reinhardtii* plastome is polyploid with ~40 copies per cell under phototrophic conditions and ~100 copies under mixotrophic conditions (Lau et al. 2000), an important early step in the analysis of putative transformant lines is to confirm that the GOI has inserted into the plastome and that all copies carry the GOI—i.e., the lines are homoplasmic (Purton 2007). We have devised a PCR strategy involving three primers (indicated in Fig. 1, with details in Table S1) to confirm both integration and homoplasmy in a single step. Primers F1 and R1 generate a 0.88-kb PCR product from the untransformed TN72 plastome, whereas in the transplastomic genome, a larger band (e.g., 1.13 kb for pSRSapI transformants) is generated by primers F1 and R2, with primer R2 designed to the promoter/5′UTR element used in each vector and primer F1 outside of the left-hand region of homology carried on each vector (thereby preventing amplification from any free plasmid or plasmid integrated into the nuclear genome). Heteroplasmic lines containing some untransformed plastome copies would be expected to give rise to both PCR products.

**Fig. 3:**
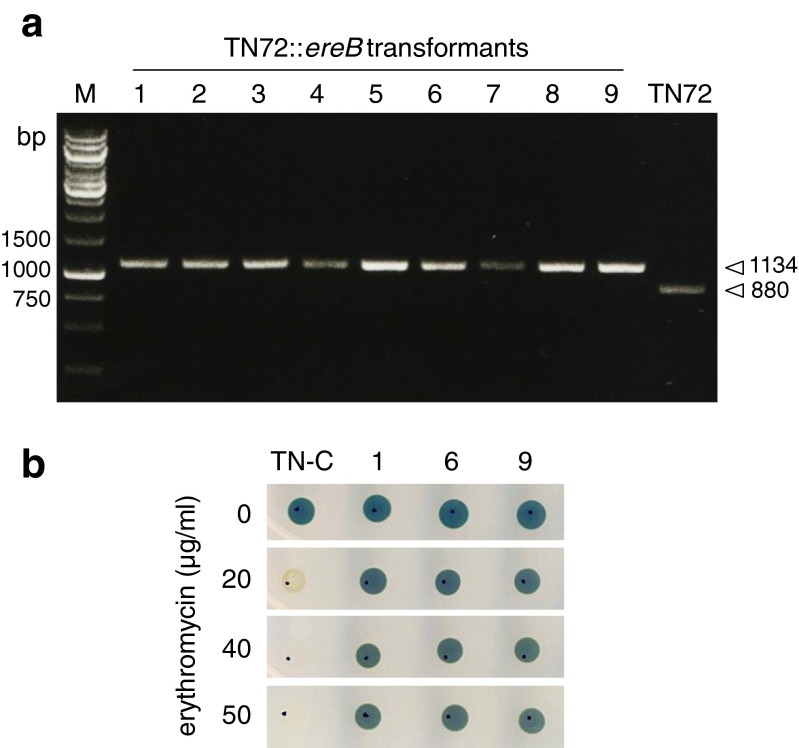
PCR confirmation of GOI integration and homoplasmy of pSRSapI-ereB transformants, and demonstration of erythromycin resistance. **a** PCR was performed using genomic DNA from transformants that had been streaked once only on HSM agar after colony picking. Primers were F1, R1, and R2 (*psaA*). The presence of a 1134-bp band confirms the successful integration of *ereB*, whereas the absence of the 880-bp band that arises from the untransformed copies of the TN72 plastome indicates that the transformant lines are homoplasmic. *M* = DNA ladder of size markers. **b** Growth of the control transformant lacking *ereB* (*TN-C*) and three representative *ereB* transformants on TAP agar plates containing increasing concentrations of erythromycin

Initial transformation experiments using pSRSapI to express a test GOI (the *E. coli ereB* gene encoding erythromycin resistance (Arthur and Courvalin 1986)) revealed that all nine colonies that were tested contained the transgene (Fig. 3a), supporting the idea that the use of the TN72 recipient ensures acquisition of the GOI in all transformants. Furthermore, it was found that selection based on restoration of phototrophy very rapidly established homoplasmy without repeated rounds of single colony isolation. This is in contrast to that observed with the commonly used *aadA* marker for spectinomycin resistance, where several rounds of colony isolation on selective medium are required to drive the transformants to a homoplasmic state (Day and Goldschmidt-Clermont 2011). The ease with which homoplasmy is obtained is probably a reflection of both the strong selective pressure to restore the photosynthetic phenotype, and the reduced plastome copy number in colonies growing on minimal medium rather than the acetate-containing medium used for *aadA* selection (Lau et al. 2000). Growth tests of three of the transformant lines on plates containing erythromycin confirmed the functional expression of *ereB* (Fig. 3b).

### The pSRSapI vector allows efficient expression in both *C. reinhardtii* and *E. coli*

**Fig. 4:**
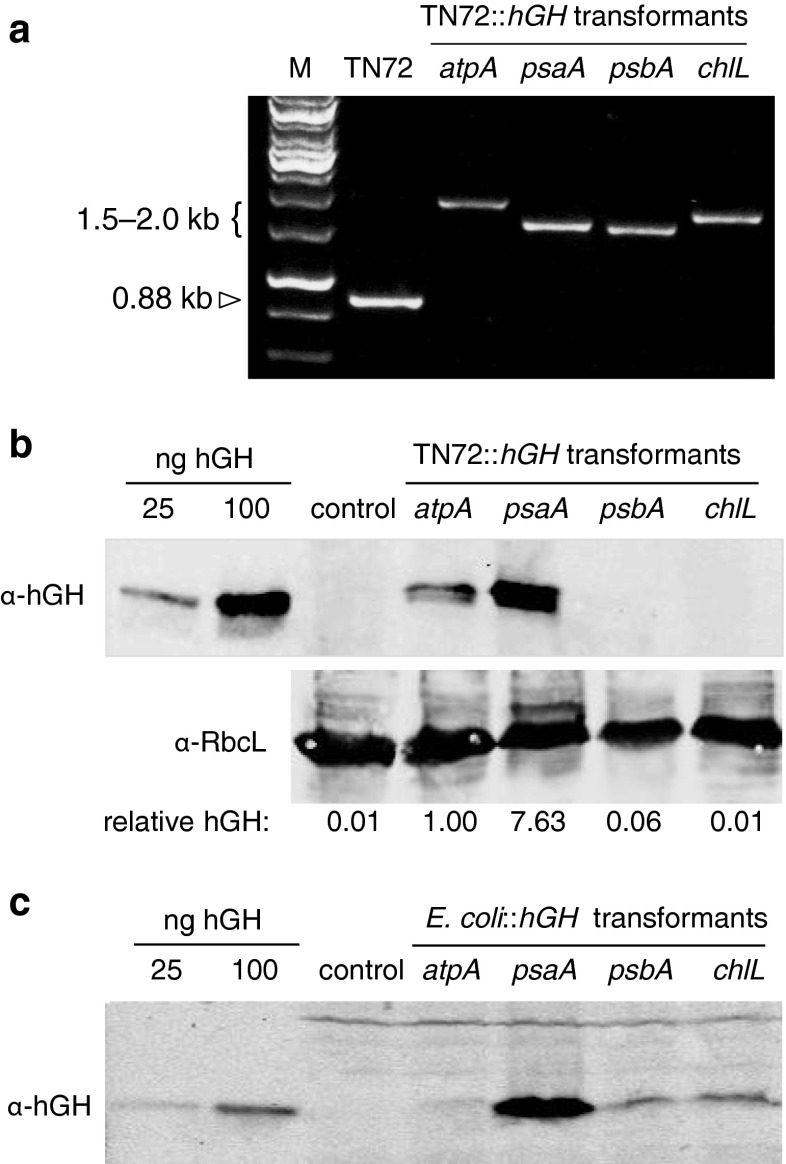
Analysis of hGH expression under the control of the different promoter/5′UTR elements. **a** PCR confirmation of gene integration and homoplasmy of *hGH* transformants. Three-primer PCR demonstrating the homoplasmic integration of hGH expression cassettes into the *C. reinhardtii* chloroplast genome. The TN72 cell line was transformed with plasmids that introduced the *hGH* into the plastome downstream of *psbH* as illustrated in Fig. 1. PCR of genomic DNA using primers F1, R1, and R3 (*hGH.R*) give a 0.88-kb product for the TN72 plastome, and a 1.5–2.0-kb product for the transgenic plastomes depending on the size of the promoter/5′UTR element. The absence of the 0.88-kb band indicates that the transformant lines are homoplasmic. *M* = DNA ladder of size markers. **b** Western blot analysis of the *C. reinhardtii* transformants. Crude protein extracts, equalized according to the optical density of the cultures, were fractionated by SDS-PAGE and probed with antibodies to hGH. *Control* = a TN72 transformant generated using the empty pSRSapI vector. Pure recombinant hGH was run alongside to aid quantification of the 22-kDa protein. An identical gel was blotted and probed with antibodies to the large subunit (*RbcL*) of the endogenous Rubisco enzyme as a loading control. Relative band intensities of hGH were quantified using an Odyssey Infrared Imaging System and scaled to give 1.000 for the *atpA* element. **c** Western blot analysis of the *E. coli* transformants. *Control* = an *E. coli* transformant generated using the empty pSRSapI vector

To compare the expression levels obtained with the four different vectors, a synthetic gene encoding human growth hormone was designed based on the codon preferences of *C. reinhardtii* chloroplast genes and cloned into each vector. Transformants were generated using TN72, and homoplasmy was established by PCR as above (Fig. 4a). A representative clone for each vector was used to assess the level of recombinant hGH in both the *C. reinhardtii* chloroplast and the *E. coli* DH5α used for the cloning. As shown in Fig. 4b, c, Western blot analysis of cell extracts from the *C. reinhardtii* and *E. coli* transformant lines using anti-hGH antibodies revealed markedly differing levels of hGH for the four different promoter/5′UTR elements. In the chloroplast, both the *psaA* and *atpA* elements gave detectable levels of hGH, with *psaA* showing a higher level as has been reported previously (Michelet et al. 2011). The protein is not detected in the *psbA* transformant lines. This is not surprising given that Rasala et al. (2011) have reported that transgenes linked to the *psbA* 5′UTR are poorly expressed due to the process of auto-attenuation of *psbA* translation which is mediated via the 5′UTR. Furthermore, Kasai et al. (2003) have reported that additional *cis* elements required for efficient *psbA* translation are located downstream of the start codon and are therefore missing in the vector. There is also no detectable hGH in the *chlL* transformant lines. This probably reflects the very low level of *chlL* expression in *C. reinhardtii*, particularly in cells grown in the light (Cahoon and Timko 2000). Furthermore, work by Cheng et al. (2005) showed that the product of a transgene (*nifH*) linked to the *chlL* promoter/5′UTR element was detectable only under conditions of anaerobic growth, suggesting that use of the *chlL* element to drive transgene expression is applicable only under such cultivation conditions. The hGH signals were normalized to the signal obtained using a control antibody raised to the large subunit of Rubisco and expressed as relative values of hGH accumulation. The hGH level from the *psaA* exon 1 element was ~7.6 times higher than from the *atpA* element (Fig. 4b). In *E. coli*, the highest level of expression is obtained also from the *psaA* construct, with levels significantly higher than those from the *psbA* and *chlL* elements. The level from the *atpA* element was barely detectable (Fig. 4c). The pSRSapI vector therefore represents a useful dual vector, able to drive transgene expression in both the *C. reinhardtii* chloroplast and in *E. coli*.

### A functional hGH is produced in the *C. reinhardtii* chloroplast

**Fig 5:**
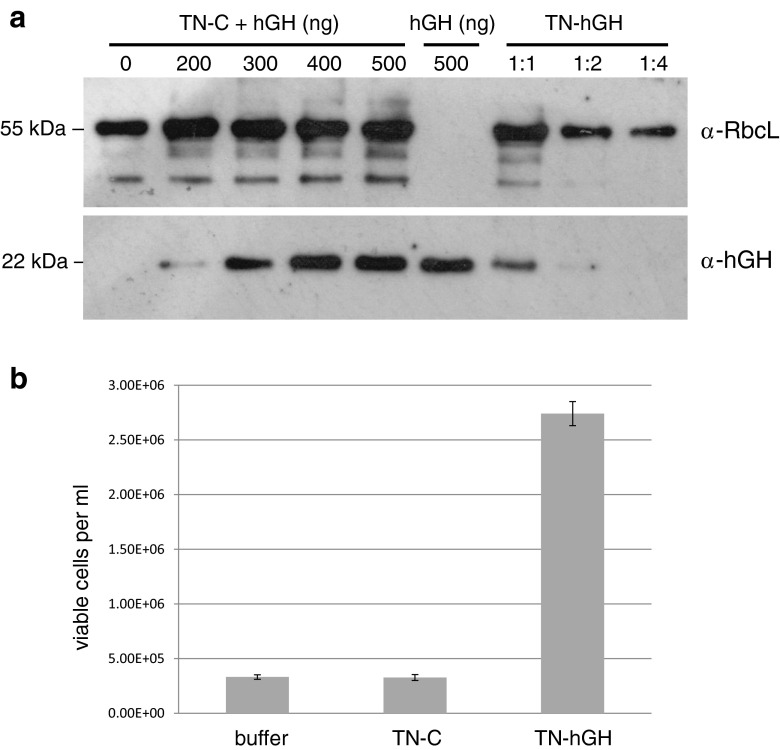
hGH quantification and activity in the TN72::pSRSapI-hGH transformant line. **a** Western blot analysis in which a cell extract from TN72::pSRSapI-hGH (*TN-hGH*) was compared with a control extract from TN72::pSRSapI (*TN-C*) spiked with known amounts of recombinant hGH. The lower part of the blot was probed with antibodies to hGH, and the upper part with antibodies to the large subunit (*RbcL*) of Rubisco as a loading control. Antibody binding was detected by ECL. **b** Determination of hGH activity using an Nb2-11 cell proliferation assay. Cell extract from the TN-hGH line (containing ~100 ng hGH) and the control line TN-C lacking hGH was added to serum-starved Nb2-11 cells at 1 × 10^5^ cells/ml and proliferation determined after 96 h. An equivalent volume of phosphate buffer was also used as a negative control. *Error bars* represent one standard deviation, *n* = 3

The transgenic line (named TN-hGH) that was generated using the pSRSapI-hGH construct was characterized further in order to assess the level of recombinant hGH produced and to determine whether the protein was functional. Figure 5a shows a Western blot in which the level of hGH in this line is compared with known amounts of a commercial sample of purified recombinant hGH. In order to mitigate against any signal differences caused by the presence of other proteins in the crude cell lysate of TN-hGH, the purified hGH was added to an equivalent crude lysate from a control transformant line (TN-C) generated using the pSRSapI vector only (Fig. 5a). Comparison of the level of hGH in the TN-hGH and spiked TN-C samples indicates that the hGH is present at ~25 μg/ml in the final TN-hGH lysate. This represents approximately 0.5 mg hGH per liter of *C. reinhardtii* culture.

To determine whether the hormone is biologically active, a proliferation assay was carried out using the Nb2-11 rat lymphoma cell line. The replication of Nb2-11 cells in suspension culture is specifically stimulated by lactogenic hormones, and by hGH which is known to have lactogenic activity (Tanaka et al. 1980; Bulatov et al. 1996). This was confirmed using the commercial hGH which showed a dose-dependent effect of the hormone on the proliferation of the cell line (Fig. S5). This proliferation was also seen when a cell extract from TN-hGH containing approximately 100 ng of hGH was added to the Nb2-11 cells, thereby confirming its biological activity. As shown in Fig. 5b, an increase in cell number of approximately eightfold was obtained after 96 h using the TN-hGH extract when compared to an equivalent extract from the control strain TN-C, or to buffer only.

## Discussion

Currently, the industrial biotechnology sector is almost exclusively based around the use of heterotrophic platforms (bacteria, yeasts, mammalian, and insect cells) for the biosynthesis of pharmaceutical proteins, bioactive metabolites, or other high-value products (Murphy 2012). Nevertheless, the ever-increasing growth of the global bioeconomy and the need for sustainable alternatives to petrochemical-based products is catalyzing interest in the exploitation of alternative cell factories, including photosynthetic microalgae such as *C. reinhardtii* (Wijffels et al. 2013). Recent successes in the biosynthesis of recombinant therapeutics in the *C. reinhardtii* chloroplast have underlined the potential of this alga as a commercial platform (Rasala and Mayfield 2015), and highlight the need for further development of new tools for rapid and reliable strain engineering (Scaife et al. 2015).

The recipient strain and vectors described here provide a low-cost and simple system for creating a transgenic line within a few weeks. Transformation of the TN72 strain is achieved by agitation with glass beads rather than the more technically complex particle bombardment method, and the strong selection for restoration of photosynthesis means that stable, homoplasmic lines are obtained without repeated rounds of selection. Importantly, the system guarantees that every selected colony contains the GOI since the deletion of the essential PSII gene in TN72 prevents spontaneous mutation to phototrophic growth, and the deletion of the genomic region between *psbH* and the site of GOI insertion ensures that restoration of *psbH* is always accompanied by insertion of the GOI. Our finding that the *psaA* promoter/5′UTR gives high levels of expression in the chloroplast is in agreement with the findings of Michelet et al. (2011), who reported that the same *psaA* element gave higher levels of their two recombinant proteins than elements from *atpA* and *psbA*. This identifies pSRSapI as the best of our expression vectors. Furthermore, the observation that the *psaA* promoter/5′UTR element used in pSRSapI is able to drive expression in *E. coli* provides a useful tool for testing the synthesis and function of the recombinant protein prior to generation of *C. reinhardtii* transformants. We speculate that the difference we see in *E. coli* expression levels for the different chloroplast gene elements is, in part, due to differences in the efficiency of translation initiation in *E. coli*. Of the four genes, the *psaA* sequence immediately upstream of the translational start most closely matches the *E. coli* Shine-Dalgarno consensus sequence for ribosome binding (Fig. S6). In contrast, a weaker match is seen for *chlL* and *psbA*, and no match is seen in the *atpA* 5′UTR. Furthermore, efficient transcription from the *psaA* promoter probably also contributes to the overall expression in *E. coli*. The 5′ end of the *psaA* exon 1 mRNA was determined by Choquet et al. (1988), and Turmel et al. (1995) predicted that a TATAAT sequence within the *psaA* exon 1 promoter region corresponded to the –10 element; this exact consensus sequence is in fact preceded by TGn, correlating to the consensus extended –10 element that is thought to contribute to promoter strength (Hook-Barnard and Hinton 2007). Promoter prediction software, softberry BPROM, indicates a possible –35 element (TTGTAA) upstream of the –10 element. These data predict that the *psaA* exon 1 promoter would give strong transcription initiation in *E. coli*. While expression in *E. coli* is a desirable feature of pSRSapI, it can also present problems when cloning genes that are deleterious to the bacterium. To address this, we have recently developed a modified version of the vector carrying an engineered chloroplast tRNA gene. The cloning of synthetic genes carrying internal UGA stop codons results in premature translation termination in *E. coli*, but in the chloroplast, the tRNA recognizes the UGA as a tryptophan codon (Young and Purton 2016).

As a test of our system, we demonstrated the functional expression of a native bacterial gene (*ereB*) and of a codon-optimized synthetic gene encoding human growth hormone (hGH). Recombinant hGH is used to treat growth hormone deficiency, Turner syndrome, and several other conditions (Kirk 2012). Glycosylation of hGH is not required for biological activity, and therefore recombinant production in prokaryotic expression systems such as *E. coli* is preferred (Sockolosky and Szoka 2013). The hormone has also been expressed successfully in the tobacco chloroplast where it was shown to fold correctly, forming the two disulfide bonds within the 22-kDa molecule (Staub et al. 2000). This is in contrast to expression in the *E. coli* cytoplasm where hGH tends to accumulate within inclusion bodies that require solubilization and refolding (Sockolosky and Szoka 2013). The *C. reinhardtii* chloroplast also possesses the machinery for the correct folding of recombinant proteins and the formation of multiple disulfide bonds (Tran et al. 2013b), and our finding that hGH is recovered in a soluble and active form further supports this. However, it should be noted that previous studies have indicated that the presence of the disulfide bonds in hGH is not specifically required for biological activity (Kim et al. 2013). Further improvement in the yield of hGH could be achieved by introducing into the transgenic line the nuclear mutation *raa-L121G*. This mutation blocks the *trans*-splicing of *psaA-exon1* mRNA and has been shown to greatly increase expression of transgenes fused to the *psaA* promoter/5′UTR (Michelet et al. 2011). Additionally, Ferreira-Camargo et al. (2015) have reported that the inclusion of selenocystamine in the growth medium improves the accumulation of proteins containing disulfide bonds and could therefore also increase hGH yield.

## Electronic supplementary material

ESM 1 (PDF 1768kb)

## References

[CR1] Arthur M, Courvalin P (1986). Contribution of two different mechanisms to erythromycin resistance in *Escherichia coli*. Antimicrob Agents Chemother.

[CR2] Barrera DJ, Rosenberg JN, Chiu JG, Chang YN, Debatis M, Ngoi SM, Chang JT, Shoemaker CB, Oyler GA, Mayfield SP (2015). Algal chloroplast produced camelid VH H antitoxins are capable of neutralizing botulinum neurotoxin. Plant Biotechnol J.

[CR3] Bateman JM, Purton S (2000). Tools for chloroplast transformation in *Chlamydomonas*: expression vectors and a new dominant selectable marker. Mol Gen Genet.

[CR4] Boynton JE, Gillham NW, Harris EH, Hosler JP, Johnson AM, Jones AR, Randolph-Anderson BL, Robertson D, Klein TM, Shark KB (1988). Chloroplast transformation in *Chlamydomonas* with high velocity microprojectiles. Science.

[CR5] Braun-Galleani S, Baganz F, Purton S (2015). Improving recombinant protein production in the *Chlamydomonas reinhardtii* chloroplast using vivid Verde fluorescent protein as a reporter. Biotechnol J.

[CR6] Bulatov AA, Osipova TA, Raevskaya GV (1996). Proliferative response of cultured Nb2 rat lymphoma cells to human serum prolactin and growth hormone. Bull Exp Biol Med.

[CR7] Cahoon AB, Timko MP (2000). *Yellow-in-the-dark* mutants of *Chlamydomonas* lack the CHLL subunit of light-independent protochlorophyllide reductase. Plant Cell.

[CR8] Chen HC, Melis A (2013). Marker-free genetic engineering of the chloroplast in the green microalga *Chlamydomonas reinhardtii*. Plant Biotechnol J.

[CR9] Cheng Q, Day A, Dowson-Day M, Shen GF, Dixon R (2005). The *Klebsiella pneumoniae* nitrogenase Fe protein gene (*nifH*) functionally substitutes for the *chlL* gene in *Chlamydomonas reinhardtii*. Biochem Biophys Res Commun.

[CR10] Choquet Y, Goldschmidt-Clermont M, Girard-Bascou J, Kuck U, Bennoun P, Rochaix J-D (1988). Mutant phenotypes support a *trans*-splicing mechanism for the expression of the tripartite *psaA* gene in the *C. reinhardtii* chloroplast. Cell.

[CR11] Choquet Y, Wollman FA (2002). Translational regulations as specific traits of chloroplast gene expression. FEBS Lett.

[CR12] Davies DR, Plaskitt A (1971). Genetical and structural analyses of cell-wall formation in *Chlamydomonas reinhardi*. Genetical Res.

[CR13] Day A, Goldschmidt-Clermont M (2011). The chloroplast transformation toolbox: selectable markers and marker removal. Plant Biotechnol J.

[CR14] Dreesen IA, Charpin-El Hamri G, Fussenegger M (2010). Heat-stable oral alga-based vaccine protects mice from *Staphylococcus aureus* infection. J Biotechnol.

[CR15] Eberhard S, Drapier D, Wollman FA (2002). Searching limiting steps in the expression of chloroplast-encoded proteins: relations between gene copy number, transcription, transcript abundance and translation rate in the chloroplast of *Chlamydomonas reinhardtii*. Plant J.

[CR16] Economou C, Wannathong T, Szaub J, Purton S (2014). A simple, low cost method for chloroplast transformation of the green alga *Chlamydomonas reinhardtii*. Methods Mol Biol.

[CR17] Esposito D, Hicks AJ, Stern DB (2001). A role for initiation codon context in chloroplast translation. Plant Cell.

[CR18] Ferreira-Camargo LS, Tran M, Beld J, Burkart MD, Mayfield SP (2015). Selenocystamine improves protein accumulation in chloroplasts of eukaryotic green algae. AMB Express.

[CR19] Gangl D, Zedler JA, Włodarczyk A, Jensen PE, Purton S, Robinson C (2015). Expression and membrane-targeting of an active plant cytochrome P450 in the chloroplast of the green alga *Chlamydomonas reinhardtii*. Phytochemistry.

[CR20] Gimpel JA, Hyun JS, Schoepp NG, Mayfield SP (2015). Production of recombinant proteins in microalgae at pilot greenhouse scale. Biotechnol Bioeng.

[CR21] Goldschmidt-Clermont M (1991). Transgenic expression of aminoglycoside adenine transferase in the chloroplast: a selectable marker of site-directed transformation of chlamydomonas. Nucleic Acids Res.

[CR22] Gong Y, Hu H, Gao Y, Xu X, Gao H (2011). Microalgae as platforms for production of recombinant proteins and valuable compounds: progress and prospects. J Ind Microbiol Biotechnol.

[CR23] Green MR, Sambrook J (2012). Molecular cloning: a laboratory manual.

[CR24] Gregory JA, Topol AB, Doerner DZ, Mayfield S (2013). Alga-produced cholera toxin-Pfs25 fusion proteins as oral vaccines. Appl Environ Microbiol.

[CR25] Harris EH, Stern DB, Witman GB (2009). The Chlamydomonas sourcebook.

[CR26] Hook-Barnard IG, Hinton DM (2007). Transcription initiation by mix and match elements: flexibility for polymerase binding to bacterial promoters. Gene Regul Syst Bio.

[CR27] Jones CS, Luong T, Hannon M, Tran M, Gregory JA, Shen Z, Briggs SP, Mayfield SP (2013). Heterologous expression of the C-terminal antigenic domain of the malaria vaccine candidate Pfs48/45 in the green algae *Chlamydomonas reinhardtii*. Appl Microbiol Biotechnol.

[CR28] Kasai S, Yoshimura S, Ishikura K, Takaoka Y, Kobayashi K, Kato K, Shinmyo A (2003). Effect of coding regions on chloroplast gene expression in *Chlamydomonas reinhardtii*. J Biosci Bioeng.

[CR29] Kim MJ, Park HS, Seo KH, Yang HJ, Kim SK, Choi JH (2013). Complete solubilization and purification of recombinant human growth hormone produced in *Escherichia coli*. PLoS ONE.

[CR30] Kindle KL, Richards KL, Stern DB (1991). Engineering the chloroplast genome: techniques and capabilities for chloroplast transformation in *Chlamydomonas reinhardtii*. Proc Natl Acad Sci U S A.

[CR31] Kirk J (2012). Indications for growth hormone therapy in children. Arch Dis Child.

[CR32] Lau KW, Ren J, Wu M (2000). Redox modulation of chloroplast DNA replication in *Chlamydomonas reinhardtii*. Antioxid Redox Signal.

[CR33] Manuell AL, Beligni MV, Elder JH, Siefker DT, Tran M, Weber A, McDonald TL, Mayfield SP (2007). Robust expression of a bioactive mammalian protein in *Chlamydomonas* chloroplast. Plant Biotechnol J.

[CR34] Maxwell K, Johnson GN (2000). Chlorophyll fluorescence—a practical guide. J Exp Bot.

[CR35] Mayfield SP, Franklin SE, Lerner RA (2003). Expression and assembly of a fully active antibody in algae. Proc Natl Acad Sci U S A.

[CR36] Michelet L, Lefebvre-Legendre L, Burr SE, Rochaix JD, Goldschmidt-Clermont M (2011). Enhanced chloroplast transgene expression in a nuclear mutant of *Chlamydomonas*. Plant Biotechnol J.

[CR37] Murphy CD (2012). The microbial cell factory. Org Biomol Chem.

[CR38] O’Connor HE, Ruffle SV, Cain AJ, Deak Z, Vass I, Nugent JH, Purton S (1998). The 9-kDa phosphoprotein of photosystem II. Generation and characterisation of *Chlamydomonas* mutants lacking PSII-H and a site-directed mutant lacking the phosphorylation site. Biochim Biophys Acta.

[CR39] Purton S (2007). Tools and techniques for chloroplast transformation of *Chlamydomonas*. Adv Exp Med Biol.

[CR40] Purton S, Szaub JB, Wannathong T, Young R, Economou CK (2013). Genetic engineering of algal chloroplasts: progress and prospects. Rus J Plant Physiol.

[CR41] Rasala BA, Mayfield SP (2015). Photosynthetic biomanufacturing in green algae; production of recombinant proteins for industrial, nutritional, and medical uses. Photosynth Res.

[CR42] Rasala BA, Muto M, Lee PA, Jager M, Cardoso RM, Behnke CA, Kirk P, Hokanson CA, Crea R, Mendez M, Mayfield SP (2010). Production of therapeutic proteins in algae, analysis of expression of seven human proteins in the chloroplast of *Chlamydomonas reinhardtii*. Plant Biotechnol J.

[CR43] Rasala BA, Muto M, Sullivan J, Mayfield SP (2011). Improved heterologous protein expression in the chloroplast of *Chlamydomonas reinhardtii* through promoter and 5′ untranslated region optimization. Plant Biotechnol J.

[CR44] Scaife MA, Nguyen GT, Rico J, Lambert D, Helliwell KE, Smith AG (2015). Establishing *Chlamydomonas reinhardtii* as an industrial biotechnology host. Plant J.

[CR45] Sockolosky JT, Szoka FC (2013). Periplasmic production via the pET expression system of soluble, bioactive human growth hormone. Protein Expr Purif.

[CR46] Specht EA, Mayfield SP (2013). Synthetic oligonucleotide libraries reveal novel regulatory elements in *Chlamydomonas* chloroplast mRNAs. ACS Synth Biol.

[CR47] Staub JM, Garcia B, Graves J, Hajdukiewicz PT, Hunter P, Nehra N, Paradkar V, Schlittler M, Carroll JA, Spatola L, Ward D, Ye G, Russell DA (2000). High-yield production of a human therapeutic protein in tobacco chloroplasts. Nat Biotechnol.

[CR48] Tanaka T, Shiu RP, Gout PW, Beer CT, Noble RL, Friesen HG (1980). A new sensitive and specific bioassay for lactogenic hormones: measurement of prolactin and growth hormone in human serum. J Clin Endocrinol Metab.

[CR49] Tran M, Henry RE, Siefker D, Van C, Newkirk G, Kim J, Bui J, Mayfield SP (2013). Production of anti-cancer immunotoxins in algae: ribosome inactivating proteins as fusion partners. Biotechnol Bioeng.

[CR50] Tran M, Van C, Barrera DJ, Pettersson PL, Peinado CD, Bui J, Mayfield SP (2013). Production of unique immunotoxin cancer therapeutics in algal chloroplasts. Proc Natl Acad Sci U S A.

[CR51] Tran M, Zhou B, Pettersson PL, Gonzalez MJ, Mayfield SP (2009). Synthesis and assembly of a full-length human monoclonal antibody in algal chloroplasts. Biotechnol Bioeng.

[CR52] Turmel M, Choquet Y, Goldschmidt-Clermont M, Rochaix J-D, Otis C, Lemieux C (1995). The *trans*-spliced intron 1 in the *psaA* gene of the *Chlamydomonas* chloroplast: a comparative analysis. Curr Genet.

[CR53] Werner R, Mergenhagen D (1998). Mating type determination of *Chlamydomonas reinhardtii* by PCR. Plant Mol Biol Rep.

[CR54] Wijffels RH, Kruse O, Hellingwerf KJ (2013). Potential of industrial biotechnology with cyanobacteria and eukaryotic microalgae. Curr Opin Biotechnol.

[CR55] Wingler A, Mares M, Pourtau N (2004). Spatial patterns and metabolic regulation of photosynthetic parameters during leaf senescence. New Phytol.

[CR56] Yoon SM, Kim SY, Li KF, Yoon BH, Choe S, Kuo MM (2011). Transgenic microalgae expressing *Escherichia coli* AppA phytase as feed additive to reduce phytate excretion in the manure of young broiler chicks. Appl Microbiol Biotechnol.

[CR57] Young REB, Purton S (2014). Cytosine deaminase as a negative selectable marker for the microalgal chloroplast: a strategy for the isolation of nuclear mutations that affect chloroplast gene expression. Plant J.

[CR58] Young REB, Purton S (2016) Codon reassignment to facilitate genetic engineering and biocontainment in the chloroplast of *Chlamydomonas reinhardtii*. Plant Biotechnol J [Epub ahead of print]. doi:10.1111/pbi.12490 10.1111/pbi.12490 PMC5102678 26471875

[CR59] Zedler JAZ, Gangl D, Hamberger B, Purton S, Robinson C (2015). Stable expression of a bifunctional diterpene synthase in the chloroplast of *Chlamydomonas reinhardtii*. J Appl Phycol.

