# Supplementary material for: New tools for chloroplast genetic engineering allow the synthesis of human growth hormone in the green alga Chlamydomonas reinhardtii
Source: Appl Microbiol Biotechnol. 2016 Feb 18;100:5467–77. doi: 10.1007/s00253-016-7354-6 (PMC4875957; doi:10.1007/s00253-016-7354-6)
Supplement: Supplementary file 1 — (PDF 1768kb) [file 253_2016_7354_MOESM1_ESM.pdf]

Electronic Supplementary Material
Applied Microbiology and Biotechnology

New tools for chloroplast genetic engineering allow the synthesis of human growth hormone in the green alga *Chlamydomonas reinhardtii*

Thanyanan Wannathong[#]*, Janet C. Waterhouse*, Rosanna E. B. Young*, Chloe K. Economou and Saul Purton

Algal Research Group, Institute of Structural and Molecular Biology, University College London, Gower Street, London, WC1E 6BT, United Kingdom

*These authors contributed equally to the work.

Corresponding author
Saul Purton s.purton@ucl.ac.uk

[#]Current address: Department of Biology, Faculty of Science, Silpakorn University, Nakornpathom 73000, Thailand.

Table S1 Primers used for plasmid construction and analysis of chloroplast transformants

Table S2 Promoter/5' UTR sequences from *C. reinhardtii* chloroplast genes used for vector construction

Fig. S1 pASapI and pSRSapI vector sequences

Fig. S2 The coding sequence for human growth hormone (hGH)

Fig. S3 Schematic diagram for the construction of a superior *C. reinhardtii* recipient

Fig. S4 Analysis of putative transformants in which *psbH* has been deleted

Fig. S5 Validation of the cell proliferation assay

Fig. S6 Alignment of sequences immediately upstream of the start codons of *psaA*, *chlL*, *psbA* and *atpA*

Table S1 Primers used for plasmid construction and analysis of chloroplast transformants

Primer	Sequence 5'-3'	Comments
Primers used for plasmid construction (changes made to incorporate RE sites shown in lowercase).		
atpA.F-MluI	CACTGTTCACgCgTCTCCAATATAG	Amplification of *atpA* promoter/5'UTR region to create pASapI
atpA.R-SapI	TCccaTGGAGTAgctcTTcCCATAAAAAAG	
ereB.F-NcoI	ATCGACCatggGGTTCGAAGAATGGGTC	Amplification of *ereB* coding region from pAT72 to clone into pSRSapI
ereB.R-SphI	ACCTAgcatgcTCATTATTCATAAACGACCTCAGA	
psaA.F	gttcacgcgtAAGCTTTCTTAATTCAACATTT	Amplification of *psaA* exon 1 promoter/5'UTR region to create pSRSapI
psaA.R	ATAGgctcttcTCATGGATTTCTCCTTATAATAAC	
psbA.F	gctgctacgcgtAATACTCCGAAGG	Amplification of *psbA* promoter/5'UTR region to create pPSapI
psbA.R	GGAGTAgctcttcCCATATGTTAATTTTTTTAAAG	
chlL.F	taaatatACGCGTGTCACCTCTTACC	Amplification of *chlL* promoter/5'UTR region to create pCSapI
chlL.R	CAgctcttcTCATAAAATCAAACTCCAG	
Primers used to confirm integration and homoplasmy (see Figure 1).		
F1	GTCATTGCGAAAATACTGGTGC	Primer is outside flanking region on expression vectors, so amplification occurs only if GOI integrates correctly. i.e. no amplification of free plasmid or nuclear integrated DNA
R1	CGGATGTAACTCAATCGGTAG	In combination with F1, PCR product is 0.88 kb
R2 (psaA)	CATGGATTTCTCCTTATAATAAC	In combination with F1, PCR product is 1.1 kb
R2 (atpA)	ACGTCCACAGGCGTCGTAAGC	In combination with F1, PCR product is 1.2 kb
R3 (hGH.R)	GAACGACATTGAACAATACG	In combination with F1, PCR product is 1.5–2.0 kb depending on promoter/5'UTR element used
psbH.R	GCAACAGGAACTTCTAAAGC	Used to confirm knockout of *psbH* in TN72
atpA.F	CAAGTGATCTTACCACTCAC	Used in combination with rbcL.R to confirm presence of aadA cassette. Product size is 1.1 kb
rbcL.R	CAAACTTCACATGCAGCAGC	Used in combination with atpA.F to confirm presence of aadA cassette. Product size is 1.1 kb

Table S2 Promoter/5’ UTR sequences from *C. reinhardtii* chloroplast genes used for vector construction

Promoter/ 5’UTR	*Sequence*
atpA	ACGCGTCTCCAATATAGTAGACTTTATTAGAGGCAGTGTTTATATACCATAAACGTCAAAAGTCATTTTTA<br>TAACTGGATCTCAAAATACCTATAAACCCATTGTTCTTCTCTTTTAGCTCTAAGAACAATCAATTTATAAA<br>TATATTTATTATTATGCTATAATATAAATACTATATAAATACATTTACCTTTTTATAAATACATTTACCTT<br>TTTTTTAATTTGCATGATTTTAATGCTTATGCTATCTTTTTTATTTAGTCCATAAAACCTTTAAAGGACCT<br>TTTCTTATGGGATATTTATATTTTCCTAACAAAGCAATCGGCGTCATAAACTTTAGTTGCTTACGACGCCT<br>GTGGACGTCCCCCCCTTCCCCTTACGGGCAAGTAAACTTAGGGATTTTAATGCAATAAATAAATTTGTCCT<br>CTTCGGGCAAATGAATTTTAGTATTTAAATATGACAAGGGTGAACCATTACTTTTGTTAACAAGTGATCTT<br>ACCACTCACTATTTTTGTTGAATTTTAAACTTATTTAAAATTCTCGAGAAAGATTTTAAAAATAAACTTTT<br>TTAATCTTTTATTTATTTTTTTCTTTTTT
psaA exon1	AAGCTTTCTTAATTCAACATTTTTAAGTAAATACTGTTTAATGTTATACTTTTACGAATACACATATGGTA<br>AAAAATAAAACAATATCTTTAAAATAAGTAAAAATAATTTGTAAACCAATAAAAAATATATTTATGGTATA<br>ATATAACATATGATGTAAAAAAAACTATTTGTCTAATTTAATAACCATGCATTTTTTATGAACACATAATA<br>ATTAAAAGCGTTGCTAATGGTGTAAATAATGTATTTATTAAATTAAATAATTGTTATTATAAGGAGAAATC<br>C
psbA	AATACTCCGAAGGAGGCAGTTGGCAGGCAACTGCCACTGACGTCCCGTAAGGGTAAGGGGACGTCCACTGG<br>CGTCCCGTAAGGGGAAGGGGACGTAGGTACATAAATGTGCTAGGTAACTAACGTTTGATTTTTTGTGGTAT<br>AATATATGTACCATGCTTTTAATAGAAGCTTGAATTTATAAATTAAAATATTTTTACAATATTTTACGGAG<br>AAATTAAAACTTTAAAAAAATTAACAT
chlL1	ACGCGTGTCACCTCTTACCACCTCTTACCATATTTCTATACTCCAAAGTAACTTTTTACATAAATGTCCCC<br>TCTGGGGCTGCCTCCTTCCCCTTCCCCTTCGGTATATAAATATAGGGCAAGTAAACTTAGCATAAACTTTA<br>GTTGCCCGAAGGGGTTTACATACTCCGAAGGAGGACAAATTTATTTATTGTGGTACAATAAATAAATTGTA<br>TGTAAACCCCTTTCGGGTAACTAAAGTTTATCACGGCAATAAGTTTCTGCTTACGCAGTATTATATCTGAC<br>GCAGTATTATATAAGAAGTTGGCAGGATAAAAATGTGTAAGTATGGCAATCTTTTAAAATAGTGTTCAATT<br>CATTTAAGGCAGATAAAAAGAAAAAAGTCCACAGGATTTAATTTTGAATAGTTCTCTATCAAAAAAAGGTT<br>TGCCGAACAATGTTTTTATTCCTGGAGTTTGATTTT

> pASapI chloroplast expression vector - 6622 bp

AGCTTGGCACTGGCCGTCGTTTTACAACGTCGTGACTGGGAAAACCCTGGCGTTACCCAACTTAATCGCCTTGCAGCACATCCCC
CTTTCGCCAGCTGGCGTAATAGCGAAGAGGCCCGCACCGATCGCCCTTCCCAACAGTTGCGCAGCCTGAATGGCGAATGGCGCCT
GATGCGGTATTTTCTCCTTACGCATCTGTGCGGTATTTCACACCGCATATGGTGCACTCTCAGTACAATCTGCTCTGATGCCGCA
TAGTTAAGCCAGCCCCGACACCCGCCAACACCCGCTGACGCGCCCTGACGGGCTTGTCTGCTCCCGGCATCCGCTTACAGACAAG
CTGTGACCGTCTCCGGGAGCTGCATGTGTCAGAGGTTTTCACCGTCATCACCGAAACGCGCGAGACGAAAGGGCCTCGTGATACG
CCTATTTTTATAGGTTAATGTCATGATAATAATGGTTTCTTAGACGTCAGGTGGCACTTTTCGGGGAAATGTGCGCGGAACCCCT
ATTTGTTTATTTTTCTAAATACATTCAAATATGTATCCGCTCATGAGACAATAACCCTGATAAATGCTTCAATAATATTGAAAAA
GGAAGAGTATGAGTATTCAACATTTCCGTGTCGCCCTTATTCCCTTTTTTGCGGCATTTTGCCTTCCTGTTTTTGCTCACCCAGA
AACGCTGGTGAAAGTAAAAGATGCTGAAGATCAGTTGGGTGCACGAGTGGGTTACATCGAACTGGATCTCAACAGCGGTAAGATC
CTTGAGAGTTTTCGCCCCGAAGAACGTTTTCCAATGATGAGCACTTTTAAAGTTCTGCTATGTGGCGCGGTATTATCCCGTATTG
ACGCCGGGCAAGAGCAACTCGGTCGCCGCATACACTATTCTCAGAATGACTTGGTTGAGTACTCACCAGTCACAGAAAAGCATCT
TACGGATGGCATGACAGTAAGAGAATTATGCAGTGCTGCCATAACCATGAGTGATAACACTGCGGCCAACTTACTTCTGACAACG
ATCGGAGGACCGAAGGAGCTAACCGCTTTTTTGCACAACATGGGGGATCATGTAACTCGCCTTGATCGTTGGGAACCGGAGCTGA
ATGAAGCCATACCAAACGACGAGCGTGACACCACGATGCCTGTAGCAATGGCAACAACGTTGCGCAAACTATTAACTGGCGAACT
ACTTACTCTAGCTTCCCGGCAACAATTAATAGACTGGATGGAGGCGGATAAAGTTGCAGGACCACTTCTGCGCTCGGCCCTTCCG
GCTGGCTGGTTTATTGCTGATAAATCTGGAGCCGGTGAGCGTGGGTCTCGCGGTATCATTGCAGCACTGGGGCCAGATGGTAAGC
CCTCCCGTATCGTAGTTATCTACACGACGGGGAGTCAGGCAACTATGGATGAACGAAATAGACAGATCGCTGAGATAGGTGCCTC
ACTGATTAAGCATTGGTAACTGTCAGACCAAGTTTACTCATATATACTTTAGATTGATTTAAAACTTCATTTTTAATTTAAAAGG
ATCTAGGTGAAGATCCTTTTTGATAATCTCATGACCAAAATCCCTTAACGTGAGTTTTCGTTCCACTGAGCGTCAGACCCCGTAG
AAAAGATCAAAGGATCTTCTTGAGATCCTTTTTTTCTGCGCGTAATCTGCTGCTTGCAAACAAAAAAACCACCGCTACCAGCGGT
GGTTTGTTTGCCGGATCAAGAGCTACCAACTCTTTTTCCGAAGGTAACTGGCTTCAGCAGAGCGCAGATACCAAATACTGTCCTT
CTAGTGTAGCCGTAGTTAGGCCACCACTTCAAGAACTCTGTAGCACCGCCTACATACCTCGCTCTGCTAATCCTGTTACCAGTGG
CTGCTGCCAGTGGCGATAAGTCGTGTCTTACCGGGTTGGACTCAAGACGATAGTTACCGGATAAGGCGCAGCGGTCGGGCTGAAC
GGGGGGTTCGTGCACACAGCCCAGCTTGGAGCGAACGACCTACACCGAACTGAGATACCTACAGCGTGAGCTATGAGAAAGCGCC
ACGCTTCCCGAAGGGAGAAAGGCGGACAGGTATCCGGTAAGCGGCAGGGTCGGAACAGGAGAGCGCACGAGGGAGCTTCCAGGGG
GAAACGCCTGGTATCTTTATAGTCCTGTCGGGTTTCGCCACCTCTGACTTGAGCGTCGATTTTTGTGATGCTCGTCAGGGGGGCG
GAGCCTATGGAAAAACGCCAGCAACGCGGCCTTTTTACGGTTCCTGGCCTTTTGCTGGCCTTTTGCTCACATGTTCTTTCCTGCG
TTATCCCCTGATTCTGTGGATAACCGTATTACCGCCTTTGAGTGAGCTGATACCGCTCGCCGCAGCCGAACGACCGAGCGCAGCG
AGTCAGTGAGCGAGGAAGCAATTCGAATCCGCGTTTTCTCCGTGAAAGGGAGGTGTCCTAGGCCTCTAGACGATGGGGGCTTTTT
GTTATATTTTACTAAATATATATTATAATTAAAAAAAATTGAATTGTCAATTTTTAATGTACACTTAGTTGAAAGTGCCCCTGTC
CCCTTGGCCATATTTAACAGAAGTTATTTATAACGCAGCTGTTTTTTGGAGTCTATAAATTTATAACATCAGTTACTATGGATTT
CCCTTTAGTTTTATGGCCTAGGACGTCCCCTTCCCCTTCGATGCTGGAGGCATCCTTTTACGGGACAATAAATAAATTTGTTGCC
TCGCCTATCGGCTAACAAGTTCCTTCGGAGTATATAAATATAGGATGTTAATACTGCTATAAACTTTAGTTGCCCAATATTTATA
TTAGGACGCCAGTGGCAGTGGTACCGCCACTGCCTGCTTCGCAGTATATAAATATAGGCAGTTGGCAGGCAACTGCCACTGACGT
CCTATTTTAATACTCCCAAGTTTACTTGCCTAGGCAGTTGGCAGGCAACAAATTTATTTATTGTCCACTAAAATTTATTTGCCCG
AAGGGGACGTCCACTAAAATTTATTTACCCGAAGGGGACGTCCTAATATAAATATGGGGATGTCAATGCTCCGTTAGGAAGTAAC
TAACGTTTTTCAAATAAATTTTATCCCGGAGGGAAGTAGGCAGTAGCCCGCCACTGTCATCCTTTAAGTGGATCTCTCGTCAGGC
AATTTGCTTACACCTTTAAATTAAAAATTAAATTTAAAGAAAAGTGAGCTATTAACGCGTCTCCAATATAGTAGACTTTATTAGA
GGCAGTGTTTATATACCATAAACGTCAAAAGTCATTTTTATAACTGGATCTCAAAATACCTATAAACCCATTGTTCTTCTCTTTT
AGCTCTAAGAACAATCAATTTATAAATATATTTATTATTATGCTATAATATAAATACTATATAAATACATTTACCTTTTTATAAA
TACATTTACCTTTTTTTTAATTTGCATGATTTTAATGCTTATGCTATCTTTTTTATTTAGTCCATAAAACCTTTAAAGGACCTTT
TCTTATGGGATATTTATATTTTCCTAACAAAGCAATCGGCGTCATAAACTTTAGTTGCTTACGACGCCTGTGGACGTCCCCCCCT
TCCCCTTACGGGCAAGTAAACTTAGGGATTTTAATGCAATAAATAAATTTGTCCTCTTCGGGCAAATGAATTTTAGTATTTAAAT
ATGACAAGGGTGAACCATTACTTTTGTTAACAAGTGATCTTACCACTCACTATTTTTGTTGAATTTTAAACTTATTTAAAATTCT
CGAGAAAGATTTTAAAAAATAAACTTTTTTAATCTTTTATTTATTTTTTCTTTTTT**ATGGGAAGAGC**TACT**CCATGGATCCTCTAG**
AGTCGACCTGCAGGCATGCAAGCTTGTACTCAAGCTCGTAACGAAGGTCGTGACCTTGCTCGTGAAGGTGGCGACGTAATTCGTT
CAGCTTGTAAATGGTCTCCAGAACTTGCTGCTGCATGTGAAGTTTGGAAAGAAATTAAATTCGAATTTGATACTATTGACAAACT
T**TAA**TTTTTATTTTTCATGATGTTTATGTGAATAGCATAAACATCGTTTTTATTTTTATGGTGTTTAGGTTAAATACCTAAACAT
CATTTTACATTTTTAAAATTAAGTTCTAAAGTTATCTTTTGTTTAAATTTGCCTGTCTTTATAAATTACGATGTGCCAGAAAAAT
AAAATCTTAGCTTTTTATTATAGAATTTATCTTTATGTATTATATTTTATAAGTTATAATAAAAGAAATAGTAACATACTAAAGC
GGATGTAGCGCGTTTATCTTAACGGAAGGCCAGTGGCAGTGGCGGTGCCACTGCCGAATATAAATATGGTTGAGTTGCTTAGTTT
ACCTTAGCGAAAAGAAGACTTAGCAGCTAGCCTTAACAAACAGTTTTATATTTTATGTTTGTGTTAAATAAAA**TTAAGAAACTTT**
AGCTAAAGTTTCCCAACTCATAGAAACGTCATCTAAAATTAAAGAACTGTTGTAAATTTCTAAAATGATTAATAAGAATGCTGCA
AATAAAAGGATAAATACAGCCATTAAAACAGTTGTACCCCAGCCTGGTAATACTTTACCTGCTTCTGAGTTAAGTGGACGTAATA
AAGTACCTAATGGTGTAACTAAACCAGGTTCTTGGAAGTCTGAATTTACTTTTGATGGTTTAGCTTTAGAAGTTCCTGTTGCCAT
AATTGATTAAATGAATTAAGCGTTATTAGCGCTATTTTATTTACTTTCTGTAAAAAATAAGGAAAATATTCTTCAGTGCATTCCC
TCTCAGGATTATAAATACTCTGAGGATAACGTTCTCTCGTCAAGGGGTTGCTTCTTGTGAGTATAGAAACCTACTAGCACAAGAA
ATAAATTGCATAAAAATGTATTTACCTAGGACCGCAGTAGGCAGTCCCTTTTCCCCTTCAGAACTGCCTGCTTTAAAAGAATGAA
AAAACTGCCTTGTCTGGTAAGTAAAACTCTTTAATTACTCACTAAAGACGATCTTAGAAGTTCTTTGTTCATTTTTTATTTAATA
TAATATTTGTTATATAAAAATTAAATAATTTTTAATTAATGTTTAACTTTGTAAGGACAGTTTCAAAGTGACATGAATGGCTACT
GCAAAAACGAAGTAAGTTATTCTTTCTCAGGGCAAAATTTTGAGTAGATTAATTTTGTTTAAAAATGTGGGACACAGTCGTCAAG
TCTTTTGAACTATCTAAGAGATATGTTGAAAAGAGAATAATTTTATTATTAAATGAGCTATGGAAAGTCCAGCTTTTTTCTTTAC
CTTTTTTTTATGGTTTCTTCTGTTAAGTGTAACTGGCTATTCAGTTTATGTTAGTTTTGGTCCACCTTCAAAAAAATTACGTGAT
CCTTTTGAAGAACACGAAGATTAAACAAGTTAAAAAGTACTATTTTTACAAGTGACTTCGGTGCCTCTGAGAACCCTAGTTATAG
TGATATAAAATAACTAGCTAACTACTTTATATTTTTATGAAAGTCATTTTGTCGAGCATATAAACAAAAACAAAATTGCTATACT
AGGCAGTCACAGTGCAACTGTCTCCGTCTCCTTAACCGAGAAAGGGTAAACGTCTTCGGTAAAGTAACAAACTTTAGTTATGTTA
ACTGCTTGCGAGTTAACCATTTTTTTTCCTCCGAAGGACAACAGTTGGCAGTTGCCAAACTTTAGTGGTCTAATATTTATATTAG
GCAGTTGGCAGGCAACTGCACTGACGTCCCGAAGGGGAAGGGGTTTACTTACCTCCTAACGGAGTATATAAATAGAATAAAATTT

ATTTCCTGCGCTAGCAGATTTACATACTAGGATTTTAATACTCCGAAGGAGGCAGTGGCGGTACCACTGCCACTGGCGTCCTCCT
TCCCCTTCGGGCAAATGCATTTTAGTGCCACTTAAGTTTACTTGCCTAGGCAGTTGGCAGGACGTCAGTGGCAGTGGTACCGCGA
CTGCCTATATTTATATACTCCTAAGTTTACTTGCCTAGGCAGTTGGCAGGCAACTGCCACTGACGTCCTTCCCCTTCCCCTTCGG
GACGTCCCCTTACGGGAATATAAATATTAGTGGATATTTATATACTGCGATGTTTACATACTCCGAAGGAGGAGAGCTAGCAGTT
GCCTGCCAACTGCCTAATATAAATATTGGGCAAGTAAACTTAGAATGTTTACATACTCCGAAGGAGGACGTCCCTTACGGGAATA
TAAATATTAGTGGCAGTGGTACCGCCACTGCCTCCTTCGGAGTATTAAAATCCTAGTATATAATATACCGTAAGGGACGTCCTCC
GACGGTGGCAGTGGCGGTACCACTGCCACCGGCGTCCTAATATACATATTGAAGTATTTAAACCTGTTAGCGCACGCTCTAACGA
GTCAGTAAACTTCCCTTTTGGGGCTTCTAGGCAGCGCATAAATTTTCTAGGACGAACGTCCACTGGCGTCTCGTAAGGAGCAGTG
ACAGGCCACTAATGTCCCCTTAATGGGTAAATAAATGGCTATCGTCTATCCATGAAGAGACCATATATTCCAGTAGCACCGTTAT
GATCCTCAAAGGGTAACACCATTTGTATAGTATTATGGTGAAATGCATCCCTTTCAGGGTAGATTTATATCTTACAG

1-2404: pUC8 vector
2405-3199: psbH downstream flanking element (0.80 kb).
3200-3798: atpA promoter/5'UTR (to ATG).
3796-3806: SapI cloning site (nnn/ngaagagc).
3811-3850: NcoI-HindIII polylinker.
3851-4257: rbcL 3' UTR element (stop codon at 3997-3999).
4258-4263: BssHII/MluI join (GCGCGT).
4264-6622: psbH upstream flanking element (2.36 kb)

>pSRSapI chloroplast expression vector 6317 bp

AGCTTGGCACTGGCCGTCGTTTTACAACGTCGTGACTGGGAAAACCCTGGCGTTACCCAACTTAATCGCCTTGCAGCACATCCCCCT
TTCGCCAGCTGGCGTAATAGCGAAGAGGCCCGCACCGATCGCCCTTCCCAACAGTTGCGCAGCCTGAATGGCGAATGGCGCCTGATG
CGGTATTTTCTCCTTACGCATCTGTGCGGTATTTCACACCGCATATGGTGCACTCTCAGTACAATCTGCTCTGATGCCGCATAGTTA
AGCCAGCCCCGACACCCGCCAACACCCGCTGACGCGCCCTGACGGGCTTGTCTGCTCCCGGCATCCGCTTACAGACAAGCTGTGACC
GTCTCCGGGAGCTGCATGTGTCAGAGGTTTTCACCGTCATCACCGAAACGCGCGAGACGAAAGGGCCTCGTGATACGCCTATTTTTA
TAGGTTAATGTCATGATAATAATGGTTTCTTAGACGTCAGGTGGCACTTTTCGGGGAAATGTGCGCGGAACCCCTATTTGTTTATTT
TTCTAAATACATTCAAATATGTATCCGCTCATGAGACAATAACCCTGATAAATGCTTCAATAATATTGAAAAAGGAAGAGTATGAGT
ATTCAACATTTCCGTGTCGCCCTTATTCCCTTTTTTGCGGCATTTTGCCTTCCTGTTTTTGCTCACCCAGAAACGCTGGTGAAAGTA
AAAGATGCTGAAGATCAGTTGGGTGCACGAGTGGGTTACATCGAACTGGATCTCAACAGCGGTAAGATCCTTGAGAGTTTTCGCCCC
GAAGAACGTTTTCCAATGATGAGCACTTTTAAAGTTCTGCTATGTGGCGCGGTATTATCCCGTATTGACGCCGGGCAAGAGCAACTC
GGTCGCCGCATACACTATTCTCAGAATGACTTGGTTGAGTACTCACCAGTCACAGAAAAGCATCTTACGGATGGCATGACAGTAAGA
GAATTATGCAGTGCTGCCATAACCATGAGTGATAACACTGCGGCCAACTTACTTCTGACAACGATCGGAGGACCGAAGGAGCTAACC
GCTTTTTTGCACAACATGGGGGATCATGTAACTCGCCTTGATCGTTGGGAACCGGAGCTGAATGAAGCCATACCAAACGACGAGCGT
GACACCACGATGCCTGTAGCAATGGCAACAACGTTGCGCAAACTATTAACTGGCGAACTACTTACTCTAGCTTCCCGGCAACAATTA
ATAGACTGGATGGAGGCGGATAAAGTTGCAGGACCACTTCTGCGCTCGGCCCTTCCGGCTGGCTGGTTTATTGCTGATAAATCTGGA
GCCGGTGAGCGTGGGTCTCGCGGTATCATTGCAGCACTGGGGCCAGATGGTAAGCCCTCCCGTATCGTAGTTATCTACACGACGGGG
AGTCAGGCAACTATGGATGAACGAAATAGACAGATCGCTGAGATAGGTGCCTCACTGATTAAGCATTGGTAACTGTCAGACCAAGTT
TACTCATATATACTTTAGATTGATTTAAAACTTCATTTTTAATTTAAAAGGATCTAGGTGAAGATCCTTTTTGATAATCTCATGACC
AAAATCCCTTAACGTGAGTTTTCGTTCCACTGAGCGTCAGACCCCGTAGAAAAGATCAAAGGATCTTCTTGAGATCCTTTTTTTCTG
CGCGTAATCTGCTGCTTGCAAACAAAAAAACCACCGCTACCAGCGGTGGTTTGTTTGCCGGATCAAGAGCTACCAACTCTTTTTCCG
AAGGTAACTGGCTTCAGCAGAGCGCAGATACCAAATACTGTCCTTCTAGTGTAGCCGTAGTTAGGCCACCACTTCAAGAACTCTGTA
GCACCGCCTACATACCTCGCTCTGCTAATCCTGTTACCAGTGGCTGCTGCCAGTGGCGATAAGTCGTGTCTTACCGGGTTGGACTCA
AGACGATAGTTACCGGATAAGGCGCAGCGGTCGGGCTGAACGGGGGGTTCGTGCACACAGCCCAGCTTGGAGCGAACGACCTACACC
GAACTGAGATACCTACAGCGTGAGCTATGAGAAAGCGCCACGCTTCCCGAAGGGAGAAAGGCGGACAGGTATCCGGTAAGCGGCAGG
GTCGGAACAGGAGAGCGCACGAGGGAGCTTCCAGGGGGAAACGCCTGGTATCTTTATAGTCCTGTCGGGTTTCGCCACCTCTGACTT
GAGCGTCGATTTTTGTGATGCTCGTCAGGGGGGCGGAGCCTATGGAAAAACGCCAGCAACGCGGCCTTTTTACGGTTCCTGGCCTTT
TGCTGGCCTTTTGCTCACATGTTCTTTCCTGCGTTATCCCCTGATTCTGTGGATAACCGTATTACCGCCTTTGAGTGAGCTGATACC
GCTCGCCGCAGCCGAACGACCGAGCGCAGCGAGTCAGTGAGCGAGGAAGCAATTCGAATCCGCGTTTTCTCCGTGAAAGGGAGGTGT
CCTAGGCCTCTAGACGATGGGGGCTTTTTGTTATATTTTACTAAATATATATTATAATTAAAAAAAATTGAATTGTCAATTTTTAAT
GTACACTTAGTTGAAAGTGCCCCTGTCCCCTTGGCCATATTTAACAGAAGTTATTTATAACGCAGCTGTTTTTTGGAGTCTATAAAT
TTATAACATCAGTTACTATGGATTTCCCTTTAGTTTTATGGCCTAGGACGTCCCCTTCCCCTTCGATGCTGGAGGCATCCTTTTACG
GGACAATAAATAAATTTGTTGCCTCGCCTATCGGCTAACAAGTTCCTTCGGAGTATATAAATATAGGATGTTAATACTGCTATAAAC
TTTAGTTGCCCAATATTTATATTAGGACGCCAGTGGCAGTGGTACCGCCACTGCCTGCTTCGCAGTATATAAATATAGGCAGTTGGC
AGGCAACTGCCACTGACGTCCTATTTTAATACTCCCAAGTTTACTTGCCTAGGCAGTTGGCAGGCAACAAATTTATTTATTGTCCAC
TAAAATTTATTTGCCCGAAGGGGACGTCCACTAAAATTTATTTACCCGAAGGGGACGTCCTAATATAAATATGGGGATGTCAATGCT
CCGTTAGGAAGTAACTAACGTTTTTCAAATAAATTTTATCCCGGAGGGAAGTAGGCAGTAGCCCGCCACTGTCATCCTTTAAGTGGA
TCTCTCGTCAGGCAATTTGCTTACACCTTTAAATTAAAAATTAAATTTAAAGAAAAGTGAGCTATTAACGCGTAAGCTTTCTTAATT
CAACATTTTTAAGTAAATACTGTTTAATGTTATACTTTTACGAATACACATATGGTAAAAAATAAAACAATATCTTTAAAATAAGTA
AAAATAATTTGTAAACCAATAAAAAATATATTTATGGTATAATATAACATATGATGTAAAAAAAACTATTTGTCTAATTTAATAACC
ATGCATTTTTTATGAACACATAATAATTAAAAGCGTTGCTAATGGTGTAAATAATGTATTTATTAAATTAAATAATTGTTATTATAA
GGAGAAATCCATGGGAAGAGCTACTCCATGGATCCTCTAGAGTCGACCTGCAGGCATGCAAGCTTGTACTCAAGCTCGTAACGAAGG
TCGTGACCTTGCTCGTGAAGGTGGCGACGTAATTCGTTCAGCTTGTAAATGGTCTCCAGAACTTGCTGCTGCATGTGAAGTTTGGAA
AGAAATTAAATTCGAATTTGATACTATTGACAAACTTTAATTTTTATTTTTCATGATGTTTATGTGAATAGCATAAACATCGTTTTT
ATTTTTATGGTGTTTAGGTTAAATACCTAAACATCATTTTACATTTTTAAAATTAAGTTCTAAAGTTATCTTTTGTTTAAATTTGCC
TGTCTTTATAAATTACGATGTGCCAGAAAAATAAAATCTTAGCTTTTTATTATAGAATTTATCTTTATGTATTATATTTATAAGTT

```
ATAATAAAAGAAATAGTAACATACTAAAGCGGATGTAGCGCGTTTATCTTAACGGAAGGCCAGTGGCAGTGGCGGTGCCACTGCCGA
ATATAAATATGGTTGAGTTGCTTAGTTTACCTTAGCGAAAAGAAGACTTAGCAGCTAGCCTTAACAAACAGTTTTATATTTTATGTT
TGTGTTAAATAAAATTAAGAAACTTTAGCTAAAGTTTCCCAACTCATAGAAACGTCATCTAAAATTAAAGAACTGTTGTAAATTTCT
AAAATGATTAATAAGAATGCTGCAAATAAAAGGATAAATACAGCCATTAAAACAGTTGTACCCCAGCCTGGTAATACTTTACCTGCT
TCTGAGTTAAGTGGACGTAATAAAGTACCTAATGGTGTAACTAAACCAGGTTCTTGGAAGTCTGAATTTACTTTTGATGGTTTAGCT
TTAGAAGTTCCTGTTGCCATAATTGATTAAATGAATTAAGCGTTATTAGCGCTATTTTATTTACTTTCTGTAAAAAATAAGGAAAAT
ATTCTTCAGTGCATTCCCTCTCAGGATTATAAATACTCTGAGGATAACGTTCTCTCGTCAAGGGGTTGCTTCTTGTGAGTATAGAAA
CCTACTAGCACAAGAAATAAATTGCATAAAAATGTATTTACCTAGGACCGCAGTAGGCAGTCCCTTTTCCCCTTCAGAACTGCCTGC
TTTAAAAGAATGAAAAAACTGCCTTGTCTGGTAAGTAAAACTCTTTAATTACTCACTAAAGACGATCTTAGAAGTTCTTTGTTCATT
TTTTATTTAATATAATATTTGTTATATAAAAATTAAATAATTTTTAATTAATGTTTAACTTTGTAAGGACAGTTTCAAAGTGACATG
AATGGCTACTGCAAAAACGAAGTAAGTTATTCTTTCTCAGGGCAAAATTTTGAGTAGATTAATTTTGTTTAAAAATGTGGGACACAG
TCGTCAAGTCTTTTGAACTATCTAAGAGATATGTTGAAAAGAGAATAATTTTATTATTAAATGAGCTATGGAAAGTCCAGCTTTTTT
CTTTACCTTTTTTTTATGGTTTCTTCTGTTAAGTGTAACTGGCTATTCAGTTTATGTTAGTTTTGGTCCACCTTCAAAAAAATTACG
TGATCCTTTTGAAGAACACGAAGATTAAACAAGTTAAAAAGTACTATTTTTACAAGTGACTTCGGTGCCTCTGAGAACCCTAGTTAT
AGTGATATAAAATAACTAGCTAACTACTTTATATTTTTATGAAAGTCATTTTGTCGAGCATATAAACAAAAACAAAATTGCTATACT
AGGCAGTCACAGTGCAACTGTCTCCGTCTCCTTAACCGAGAAAGGGTAAACGTCTTCGGTAAAGTAACAAACTTTAGTTATGTTAAC
TGCTTGCGAGTTAACCATTTTTTTTCCTCCGAAGGACAACAGTTGGCAGTTGCCAAACTTTAGTGGTCTAATATTTATATTAGGCAG
TTGGCAGGCAACTGCACTGACGTCCCGAAGGGGAAGGGGTTTACTTACCTCCTAACGGAGTATATAAATAGAATAAAATTTATTTCC
TGCGCTAGCAGATTTACATACTAGGATTTTAATACTCCGAAGGAGGCAGTGGCGGTACCACTGCCACTGGCGTCCTCCTTCCCCTTC
GGGCAAATGCATTTTAGTGCCACTTAAGTTTACTTGCCTAGGCAGTTGGCAGGACGTCAGTGGCAGTGGTACCGCGACTGCCTATAT
TTATATACTCCTAAGTTTACTTGCCTAGGCAGTTGGCAGGCAACTGCCACTGACGTCCTTCCCCTTCCCCTTCGGGACGTCCCCTTA
CGGGAATATAAATATTAGTGGATATTTATATACTGCGATGTTTACATACTCCGAAGGAGGAGAGCTAGCAGTTGCCTGCCAACTGCC
TAATATAAATATTGGGCAAGTAAACTTAGAATGTTTACATACTCCGAAGGAGGACGTCCCTTACGGGAATATAAATATTAGTGGCAG
TGGTACCGCCACTGCCTCCTTCGGAGTATTAAAATCCTAGTATATAATATACCGTAAGGGACGTCCTCCGACGGTGGCAGTGGCGGT
ACCACTGCCACCGGCGTCCTAATATACATATTGAAGTATTTAAACCTGTTAGCGCACGCTCTAACGAGTCAGTAAACTTCCCTTTTG
GGGCTTCTAGGCAGCGCATAAATTTTCTAGGACGAACGTCCACTGGCGTCTCGTAAGGAGCAGTGACAGGCCACTAATGTCCCCTTA
ATGGGTAAATAAATGGCTATCGTCTATCCATGAAGAGACCATATATTCCAGTAGCACCGTTATGATCCTCAAAGGGTAACACCATTT
GTATAGTATTATGGTGAAATGCATCCCTTTCAGGGTAGATTTATATCTTACAG
```

psaA promoter/5'UTR
SapI (LguI) and SphI (PaeI) sites
rbcL 3'UTR (also contains end of rbcL CDS)
psbH gene (reverse orientation)
Unique MluI site – can be used as site for insertion of additional transgene cassettes.

Fig. S1 pASapI and pSRSapI vector sequences

```
>Codon-optimised hGH
accgctcttctATGTTTCCAACAATTCCATTATCACGTTTATTTGATAATGCTATGTTACGTGCTCATCGTTTAC
ACCAATTAGCTTTTGATACTTATCAAGAATTTGAAGAAGCTTATATTCCAAAAGAACAAAAATACTCATTTTTAC
AAAATCCACAAACATCATTATGTTTTTCAGAATCAATTCCAACACCATCAAATCGTGAAGAAACACAACAAAAAT
CAAACTTAGAATTATTACGTATTTCATTATTATTAATTCAATCTTGGTTAGAACCAGTTCAATTTTTACGTTCAG
TTTTTGCTAATTCATTAGTTTATGGTGCTTCAGATTCAAATGTTTATGACTTATTAAAAGACTTAGAAGAAGGTA
TTCAAACATTAATGGGTCGTTTAGAAGATGGTTCACCACGTACAGGTCAAATTTTCAAACAAACATATTCAAAAT
TTGATACAAATTCACACAATGATGATGCTTTATTAAAAAATTATGGTTTATTATATTGTTTTCGTAAAGATATGG
ATAAAGTTGAAACATTTTTACGTATTGTTCAATGTCGTTCAGTTGAAGGTTCATGTGGTTTTTAAgcatgcgaat

>hGH protein sequence
MFPTIPLSRLFDNAMLRAHRLHQLAFDTYQEFEEAYIPKEQKYSFLQNPQTSLCFSESIPTPSNREETQQKSNLE
LLRISLLLIQSWLEPVQFLRSVFANSLVYGASDSNVYDLLKDLEEGIQTLMGRLEDGSPRTGQIFKQTYSKFDTN
SHNDDALLKNYGLLYCFRKDMDKVETFLRIVQCRSVEGSCGF-
```

Fig. S2 The coding sequence for human growth hormone (hGH), codon-optimized for the *Chlamydomonas reinhardtii* chloroplast, is shown in uppercase with the *Sap*I and *Sph*I restriction sites used for cloning into to pSRSapI, etc. highlighted in red. The hGH protein sequence is shown in blue with the four cysteine residues that form two disulphide bonds highlighted in yellow.

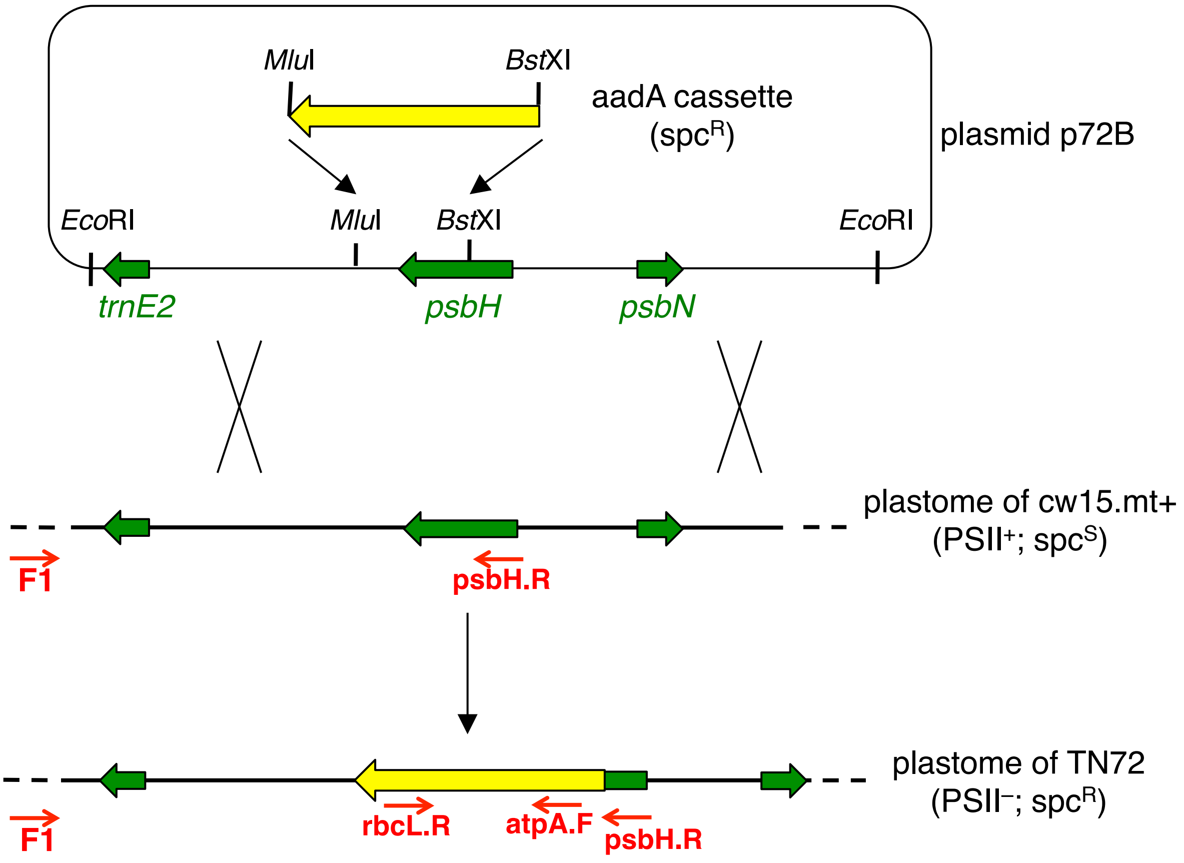

Fig. S3 Schematic diagram for the construction of a superior *C. reinhardtii* recipient. The *aadA* cassette (Goldschmidt-Clermont 1991) was cloned into plasmid p72B (Bateman and Purton 2000) to replace the region from the *Mlu*I site downstream of *psbH* to the *Bst*XI site in the middle of the gene. The resulting plasmid p72B-aadA-BM was used for glass bead transformation of the cell-wall-deficient strain *cw15*(mt+) with transformant colonies selected on TAP-agar plates containing 100 µg/ml spectinomycin as described in Economou et al. 2014.

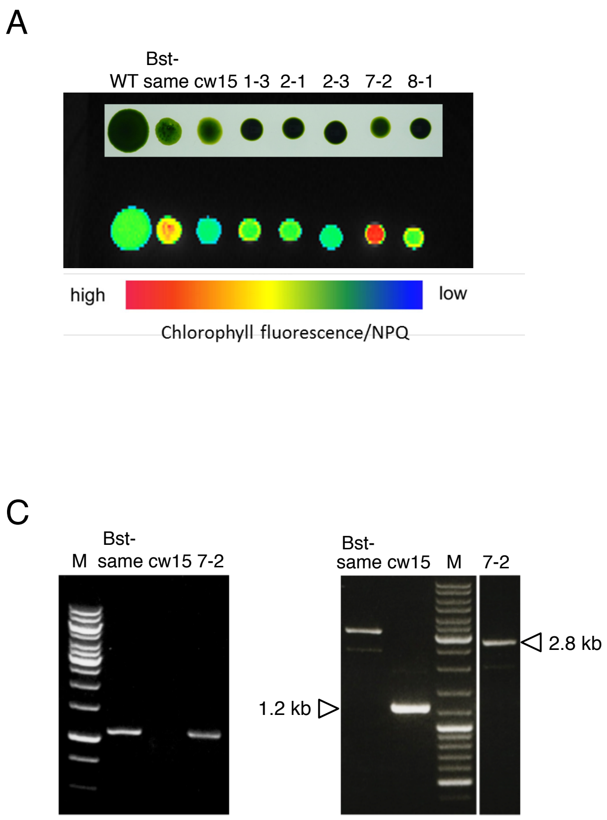

Fig. S4 **(a)** Analysis of putative transformants by chlorophyll fluorescence to identify high fluorescence mutants lacking Photosystem II as a result of the *psbH* deletion. Spectinomycin resistant lines were grown on TAP medium in dim light, and then dark-adapted for pulse amplitude modulation (PAM) fluorescence analysis (Wingler et al. 2004). False colour images reveal that line 7-2 has a high chlorophyll fluorescence, similar to that of the original *psbH*-knockout strain, Bst-same (O'Connor et al. 1998). **(b)** Growth tests confirm the phenotype of line 7-2 as unable to grow phototrophically on minimal medium (HSM) and resistant to spectinomycin and streptomycin (each at 100 µg/ml). **(c)** PCR analysis of 7-2 confirms the correct integration of the *aadA* cassette into the plastome, as indicated in Fig. S3. Left hand panel: PCR using primers atpA.F and rbcL.R that bind the *atpA* 5'UTR and *rbcL* 3'UTR respectively, amplify the *aadA* spectinomycin resistance cassette in both 7-2 and the original Bst-same strain (Economou et al. 2014). Right hand panel: primers F1 and psbH.R that bind to the region flanking the integration site and to the 3' end of *psbH*, respectively. Line 7-2 was subsequently named TN72.

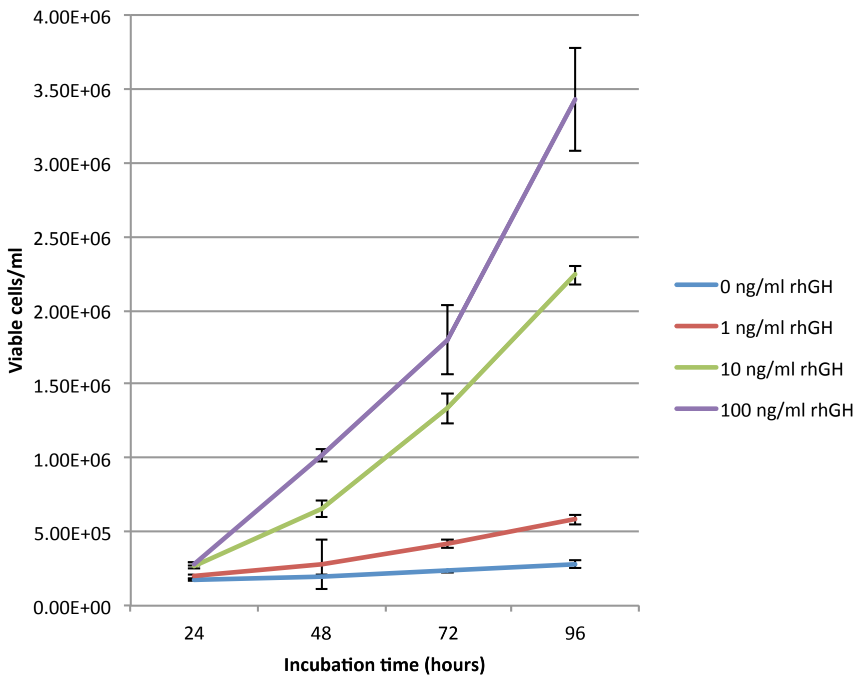

Fig. S5 Validation of the cell proliferation assay. Commercial recombinant hGH (0 to 100 ng/ml) was added to serum-starved Nb2-11 rat lymphoma cells (1×10^5 cells/ml) and viable cell number measured using the CASY® Cell Counter and Analyzer Model TT (Innovatis) at 24, 48, 72 and 96 hours. *Error bars* represent one standard deviation, *n*=3

```
      E. coli consensus:   ...GGAGGAU..A/U..AUG
                              (-10/-4) (-3)

           -30       -20       -10       +1
            .         .         .         .
psaA:   UUAAAUUAAAUAAUUGUUAUUAUAAGGAGAAAUCCAUG
chlL:   GCCGAACAAUGUUUUUAUUCCUGGAGUUUGAUUUUAUG
psbA:   UUACGGAGAAAUUAAAACUUUAAAAAAAUUAACAUAUG
atpA:   AACUUUUUUAAUCUUUUAUUUAUUUUUUCUUUUUUAUG
```

Fig. S6 Alignment of sequences immediately upstream of the start codons of *psaA*, *chlL*, *psbA* and *atpA* and identification of bases matching the Shine-Dalgarno consensus sequence for ribosome binding in *E. coli*. The *psaA* sequence shows the strongest match and *atpA* the weakest.

References

Economou C, Wannathong T, Szaub J, Purton S (2014) A simple, low cost method for chloroplast transformation of the green alga *Chlamydomonas reinhardtii*. Methods Mol Biol 1132:401–411.

Goldschmidt-Clermont M (1991) Transgenic expression of aminoglycoside adenine transferase in the chloroplast: a selectable marker of site-directed transformation of chlamydomonas. Nucleic Acids Res 19:4083–4089.

O'Connor HE, Ruffle SV, Cain AJ, Deak Z, Vass I, Nugent JH, Purton S (1998) The 9-kDa phosphoprotein of photosystem II. Generation and characterisation of *Chlamydomonas* mutants lacking PSII-H and a site-directed mutant lacking the phosphorylation site. Biochim Biophys Acta 1364:63–72.

Wingler A, Mares M, Pourtau N (2004) Spatial patterns and metabolic regulation of photosynthetic parameters during leaf senescence. New Phytologist 161:781–789.
